# Kinetics of Abacavir-Induced Remodelling of the Major Histocompatibility Complex Class I Peptide Repertoire

**DOI:** 10.3389/fimmu.2021.672737

**Published:** 2021-05-19

**Authors:** Patricia T. Illing, Andy van Hateren, Rachel Darley, Nathan P. Croft, Nicole A. Mifsud, Samuel King, Lyudmila Kostenko, Mandvi Bharadwaj, James McCluskey, Tim Elliott, Anthony W. Purcell

**Affiliations:** ^1^ Infection and Immunity Program, Department of Biochemistry and Molecular Biology, Monash Biomedicine Discovery Institute, Monash University, Clayton, VIC, Australia; ^2^ Institute for Life Sciences and Centre for Cancer Immunology, Faculty of Medicine, University of Southampton, Southampton, United Kingdom; ^3^ Department of Microbiology and Immunology, Peter Doherty Institute for Infection and Immunity, University of Melbourne, Parkville, VIC, Australia; ^4^ Nuffield Department of Medicine, University of Oxford, Oxford, United Kingdom

**Keywords:** MHC I antigen presentation, abacavir, T cells, drug hypersensitivity, immunopeptidome, tapasin, peptide selection

## Abstract

Abacavir hypersensitivity syndrome can occur in individuals expressing the HLA-B*57:01 major histocompatibility complex class I allotype when utilising the drug abacavir as a part of their anti-retroviral regimen. The drug is known to bind within the HLA-B*57:01 antigen binding cleft, leading to the selection of novel self-peptide ligands, thus provoking life-threatening immune responses. However, the sub-cellular location of abacavir binding and the mechanics of altered peptide selection are not well understood. Here, we probed the impact of abacavir on the assembly of HLA-B*57:01 peptide complexes. We show that whilst abacavir had minimal impact on the maturation or average stability of HLA-B*57:01 molecules, abacavir was able to differentially enhance the formation, selectively decrease the dissociation, and alter tapasin loading dependency of certain HLA-B*57:01-peptide complexes. Our data reveals a spectrum of abacavir mediated effects on the immunopeptidome which reconciles the heterogeneous functional T cell data reported in the literature.

## Introduction

Major histocompatibility complex class I (MHC I) molecules acquire peptide antigens in the endoplasmic reticulum (ER) and present their peptide cargo at the cell surface for scrutiny by cytotoxic T lymphocytes (CTLs). These peptide antigens are derived from the degradation of intracellular proteins along with other sources, including peptides derived from defective ribosomal products (DRiPs) produced during aborted protein synthesis (1). Therefore, the peptides presented by MHC I molecules (coined the immunopeptidome) provide an overview of cellular protein production. As such, virus infected or cancerous cells can be detected when novel peptides, such as those derived from the viral proteome or neo-epitopes generated during oncogenesis, are presented on the cell surface. Their recognition by CTLs stimulates cytotoxicity against the antigen presenting cells, facilitating elimination of the infected or transformed cells.

MHC I molecules are noted for their exceptionally high allelic polymorphism and subsequent sequence diversity. The polymorphisms that differentiate MHC I allotypes are generally concentrated within the antigen binding groove. Consequently, each MHC I allotype has a unique peptidome, containing characteristic ligands with positional biases for subsets of amino acid residues at two to four “anchor” positions of the bound peptide. As a result, the peptide repertoire displayed by MHC I molecules at the cell surface is shaped by both the peptides liberated during protein degradation as well as by polymorphisms within the antigen binding groove that dictate ligand selection and further interactions with peptide loading chaperones (2–4).

In humans, classical MHC I molecules are co-dominantly expressed from three gene loci known as Human Leukocyte Antigen (HLA)-*A*, *-B* and *-C*. In addition to expected genetic associations between different HLA-A, B, and C allotypes and susceptibility to various infectious agents (5–10) or autoimmune disorders (11–18), quite profound associations have also been made between these genes and drug hypersensitivity reactions (19). Some of these HLA associations represent the strongest of any HLA-linked responses with odds ratios (>500) for distinct hypersensitivity reactions towards the anti-epileptic carbamazepine and HLA-B*15:02, the anti-hyperuricaemic allopurinol and HLA-B*58:01, and the anti-retroviral abacavir and HLA-B*57:01 (20–22). The molecular interaction underpinning these associations is only known for abacavir, where the drug behaves as a non-peptide ligand of HLA-B*57:01, binding within the peptide-binding groove and partially satiating the unique F pocket of this HLA allotype. This effectively creates a distinct, shallower antigen binding cleft that selects novel co-occupying peptide ligands (23–25). The immunopeptidome perturbation is characterised by a switch in C-terminal anchor residue (P*Ω*) preference from “canonical” HLA-B*57:01 peptides with aromatic residues (Trp/Phe/Tyr) at their C-terminus to “abacavir-induced” peptides with smaller aliphatic residues (Ile/Leu/Val) at this position. This results in the appearance of a large number of novel HLA–abacavir–peptide complexes at the cell surface and corresponding vigorous CTL responses against drug exposed cells (25, 26). These drug-induced anti-”altered self” responses are the proposed basis of the potentially deadly abacavir hypersensitivity syndrome in HLA-B*57:01^+^ HIV patients utilising the drug as a part of their anti-retroviral regimen.

The involvement of the conventional MHC I antigen presentation pathway in the generation of abacavir-specific responses (26) suggests that novel HLA-B*57:01–peptide complexes are formed *de novo* in the ER in the presence of the drug (25). Peptide selection and the formation of stable ligand bound complexes is an intrinsic property of MHC I molecules, a process that varies in efficiency between allotypes and is enhanced *via* a macromolecular peptide loading complex (3, 27, 28). Peptide selection is thought to result from an iterative process in the ER in which MHC I molecules progressively replace low affinity peptides with those that bind with higher affinity (29, 30). A key constituent of this loading complex is the MHC I-specific co-factor tapasin, which is chiefly responsible for increasing the rate and extent of peptide loading and enhancing the discrimination that occurs between peptides (31–34). As such, tapasin ensures that MHC I preferentially assembles with high affinity peptides conferring increased expression at the cell surface (3, 35, 36). Although abacavir has a clear impact on the final immunopeptidome, it is unknown how abacavir interacts with this editing process.

Previous studies have indicated that the sensitisation of HLA-B*57:01^+^ targets for killing by drug responsive T cells is dependent on a functional peptide loading complex and required *de novo* generation of HLA–drug–peptide complexes (25, 26). However, a small subset of drug-responsive CTLs are reported to target HLA-B*57:01^+^ cells almost immediately upon abacavir exposure, raising the possibility that abacavir might exert a direct effect on HLA-B*57:01 molecules expressed at the cell surface (37). We therefore sought to i) define the kinetics of abacavir-induced changes in the HLA-B*57:01 immunopeptidome and to assess whether this correlates with *de novo* HLA-B*57:01 complex formation and immunogenicity; and ii) characterise the impact of abacavir on the association and dissociation of specific canonical and abacavir induced peptide ligands of HLA-B*57:01 using an *in vitro* peptide loading assay. In doing so, we generated a model in which abacavir allows the introduction of novel self-peptides into the immunopeptidome of HLA-B*57:01 that are not normally selected by HLA-B*57:01, as well as enhancing presentation of a subset of constitutive ligands by augmenting their association to, and reducing their dissociation from, HLA-B*57:01. The diverse effect that abacavir has on the selection of the B*57:01 peptide repertoire likely underpins the heterogeneity in response kinetics of different abacavir-induced T cell clones (37).

## Materials and Methods

### Ethics and Sample Collection

HLA-B*57:01^+^ healthy individuals (n = 3) were recruited for the study. Ethics approval was granted by both Monash University (HREC 4717 & 2297) and the Australian Bone Marrow Donor Registry (2013/04). Informed consent was obtained from all subjects. Peripheral blood samples were collected in heparinised vacutainer tubes and peripheral blood mononuclear cells (PBMCs) isolated by Ficoll–Paque (GE Healthcare) and density gradient centrifugation and cryopreserved until required.

### Cell Lines

The C1R.B*57:01 cell line is a HLA-B*57:01 transfectant of the Class I reduced C1R cell line [expresses low levels of endogenous HLA-A2 and -B35, and normal levels HLA-Cw4 (38, 39)] and has been previously used in studies of interactions between abacavir and HLA-B*57:01 (25, 26). HLA expression was maintained in long-term culture through addition of Geneticin, 0.5 mg/ml (G418; Life Technologies). C1R.B*57:01 were cultured in RF10 [RPMI 1640 (Life Technologies) supplemented with 10% foetal calf serum (FCS; Sigma), 7.5 mM HEPES (MP Biomedicals), 100 U/ml Pen Strep (benzyl–penicillin/streptomycin, Life Technologies), 2 mM L-glutamine (MP Biomedicals), 76 μM *β*-mercaptoethanolamine (Sigma), and 150 μM non-essential amino acids (Life Technologies)] at 37°C with 5% CO_2_. HLA-expression phenotype was confirmed by flow cytometry using W6/32 (pan HLA class I, produced in-house from hybridoma) or 3E12 [HLA-B57 specific (40)] antibodies.

The 721.220 and tapasin transfectant 721.220.tapasin are previously described (3, 27) and were maintained in RPMI (Sigma) with 10% foetal bovine serum (Globepharm), 2 mM L-glutamine (Sigma), and 10 mM HEPES (Lonza) at 37°C with 5% CO_2_. HLA-B*57:01 cDNA encoding amino acids −24 to 338 were cloned into pMCFR containing a puromycin selection marker. 721.220 and 721.220.tapasin cells were transfected using Nucleofection, program A-23, solution T (Amaxa) with 2 µg purified plasmid (Qiagen). Stable, polyclonal transfectants were selected with 1.5 µg/ml puromycin (Sigma) and/or 1 mg/ml G418 (Invitrogen) for tapasin expressing cell lines. The HLA-expression phenotype of the cells was confirmed by flow cytometry using W6/32 antibody.

### Isolation of HLA Ligands

C1R.B*57:01 cells were grown to high density in RF10 containing 0.5 mg/ml G418 with the addition of 35 µM abacavir (Ziagen^®^ tablets, GlaxoSmithKline) for the final 2, 4, 6, 12, or 16 h of culture, or with abacavir maintained at 35 µM throughout culture, or without abacavir. Cells were harvested by centrifugation (500 g, 5 min, 4°C), washed twice in chilled PBS and pellets of 10^8^ cells snap frozen by submersion in liquid nitrogen. All samples were generated in triplicate and stored at −80°C prior to HLA extraction. For cell lysis prior to isolation of HLA molecules, cell pellets were resuspended in 5 ml of a mild lysis buffer {0.5% IGEPAL 630 [Sigma], 50 mM Tris pH 8, 150 mM NaCl [Merck-Millipore] and protease inhibitors [Complete Protease Inhibitor Cocktail Tablet (1 tablet per 50 ml solution); Roche Molecular Biochemicals, Switzerland]} and incubated for 45 min at 4°C with slow rolling to mix. Lysates were cleared by centrifugation at 16,000 *g* for 20 min at 4°C in a bench top centrifuge.

Immunoaffinity purification of HLA-peptide complexes from lysates was performed using the pan class I antibody W6/32 (produced in-house from hybridoma) coupled to a protein A affinity resin (Repligen) as described previously (41). Complexes were dissociated using 10% acetic acid (Sigma) and fractionated by reversed-phase high performance liquid chromatography (RP-HPLC) using an ÄKTAmicro™ HPLC system (GE Healthcare) equipped with a monolithic C_18_ RP-HPLC column (4.6 mm i.d. × 50 mm length, Chromolith Speed Rod, Merck-Millipore) and running a mobile phase consisting of buffer A [0.1% trifluoroacetic acid (TFA, Thermo Scientific)] and buffer B [80% acetonitrile (ACN, Fisher Scientific), 0.1% TFA] as described elsewhere (42). UV absorbance at 215 nm monitored the elution of material from the column and the area under the curve for the *β*_2_-microglobulin (*β*_2_m) peak used as a measure of the purified HLA within the sample. Peptide containing fractions were concatenated into three fraction pools, concentrated using a speed vacuum concentration system (LABCONCO) and reconstituted with 30 µl with 0.1% formic acid (FA, Thermo Scientific) prior to LC-mass spectrometry (MS) analysis.

### Targeted Mass Spectrometric Detection and Quantitation of HLA Ligands

Liquid chromatography-multiple reaction monitoring (LC-MRM)-MS experiments utilised a Tempo nanoLC (Eksigent) autosampler and cHiPLC nanoflex (Eksigent) paired to a SCIEX QTRAP 5500 mass spectrometer. 10 µl samples were loaded onto a cHiPLC trap column (ChromXP C_18_-CL column 0.5 mm × 200 µm i.d., 3 µm particle size, nominal pore size 120 Å) at 5 µl/min in 2% ACN, 0.1% FA for 10 min. An analytical cHiPLC column (ChromXP C_18_-CL 15 cm × 75 µm i.d., 3 µm particle size, nominal pore size 120 Å) was switched in line, and the peptides eluted at 300 nl/min over a gradient of buffer A (0.1% FA) and B (98% ACN, 0.1% FA): 0–1 min 2% B, 1–3 min 2–10% B, 3–40 min 10–35.5% B, 40–45 min 35.5–80% B, 45–50 min hold at 80% B, 50–53 min 80–2% B, re-equilibration at 2% B for 7 min.

The QTRAP 5500 was operated in MRM mode in unit resolution for Q1 and Q3, with any MRM transition exceeding 600 counts triggering an Enhanced Product Ion (EPI) scan. We previously analysed the immunopeptidome of HLA class I from C1R.B*57:01 (untreated or constant 35 µM abacavir treatment) by LC-tandem mass spectrometry (MS/MS) using an SCIEX 5600^+^ TripleTOF system (25). LC-MS/MS data were searched against the reviewed human proteome (UniProt/SwissProt accessed April 2016) using ProteinPilot™ software v4.5 considering biological modifications and employing a decoy database for false discovery rate (FDR) analysis. Peptides for quantitation were chosen from these data and MRM parent-product ion transitions were designed from the prominent product ions observed in the experimental spectra of selected peptides. Detection of these transitions overlapping at a particular retention time (RT) was used as an indicator of peptide presence. Peptide identity was validated either through comparison of transition hierarchy and RT to synthetic peptides, or, in the absence of synthetic peptide data, assignment of the fragmentation spectra observed in the linked EPI scan when searched against the list of candidate peptides using Protein Pilot™ software v5.0. Transitions were also designed for the detection of abacavir, a singly charged ion of mass:charge ratio (m/z) +287.2 with fragment ions of 190.9 (Collision energy [CE] 30), 174.0 (CE 45), 164.1 (CE 39), and 150.0 (CE 46).

A relative measure of peptide or abacavir abundance within the immunopeptidome was calculated as the total area under the curve for the detected transitions using Skyline software 64 bit 4.1.0.18169 [MacCoss Laboratory (43)] normalised to the total yield of class I HLA complexes (*i.e.* normalised to the area of the *β*_2_m peak observed on RP-HPLC separation of the immunoaffinity eluate). Mean abundance was calculated from three replicates for each time point and normalised to the maximum mean detection of the peptide/abacavir. To categorise peptides based on abacavir impact, linear regression was performed over t = 0 (untreated) to t = 16 h abacavir exposure using GraphPad Prism 7.01 (GraphPad Software, San Diego, California, USA). Peptides were defined as abacavir inhibited (slope < −0.02), minimal impact (slope magnitude < 0.02), abacavir facilitated (slope > 0.02, abundance > 0 at t = 0 in at least two of three replicates), abacavir dependent (slope > 0.02, abundance = 0 at t = 0 in at least two of three replicates). Although not matching the slope criteria peptides, KTFTTQETI and KTIETSPSL were classified as abacavir facilitated because the signal observed on constant treatment with abacavir was more than twice that observed in the absence of abacavir treatment. The mass spectrometry proteomics data have been deposited to the ProteomeXchange Consortium *via* the PRIDE (44) partner repository with the dataset identifier PXD024331.

### Comparison of the Cellular Proteome Using Label Free Quantitation in LC-MS/MS

Triplicate cultures of C1R.B*57:01 in RF10 +/− 35 µM abacavir were incubated at 37°C, 5% CO_2_ for 48 h. Cells were then harvested by centrifugation (500 g, 5 min, room temperature), washed in PBS and pellets of 7 × 10^6^ cells snap frozen on dry ice in Eppendorf^®^ LoBind microcentrifuge tubes and stored at −80°C until time of lysis. Cell pellets were lysed by resuspension in 100 µl 4% Sodium dodecyl sulphate (SDS, Sigma), 0.1 M Tris-HCl pH 7.6 (Merck-Millipore), 0.1 M Dithiothreitol (DTT, Sigma) followed by incubation at 95°C with shaking (1,200 rpm, Eppendorf Thermomixer Comfort). Insoluble material was removed by centrifugation (16,000 *g*, 10 min, RT) and supernatants harvested. Protein concentration was determined using a standard Bradford assay (Expedeon). 15 µl lysate (average 300 µg protein) was subjected to trypsin digestion using a FASP™ Protein Digestion kit (Expedeon) using a 10 kDa cut-off spin filter (Pall Corporation, USA) and proteomics grade trypsin from porcine pancreas (Sigma-Aldrich, USA). 10 µl digested peptides were acidified by addition of FA to a final concentration of 1% and desalted using OMIX C18 tips (Agilent Technologies), eluting in 50% ACN, 0.1% FA. ACN was removed using a speed vacuum concentration system (LABCONCO, USA) and samples resuspended in 15 µl 0.1% FA for mass spectrometry analysis. LC-MS/MS was performed using a Dionex UltiMate 3000 RSLCnano system coupled to a QExactive Plus mass spectrometer (Thermo Scientific). 6 µl of digest samples were loaded onto an Acclaim^®^ PepMap100 20 mm C18 Nano-Trap column (100 μm i.d, 5 μm particle size, 100 Å pore size) at 15 µl/min in 2% ACN, 0.1% FA. Peptides were eluted from the trap column and separated over a 50 cm analytical Acclaim^®^ RSLC PepMap RSLC 50 cm C18 Nano column (75 μm i.d., 2 μm particle size, 100 Å pore size), equilibrated in 97.5% buffer A (0.1% FA)/2.5% buffer B (80% ACN, 0.1% FA) at 250 nl/min using the following gradient conditions: 2.5–7.5% B over 1 min, 7.5–40% B over 120 min, 40–99% B over 5 min, 6 min hold at 99% B, followed by re-equilibration in 2.5% B for 20 min. Peptide detection was performed using a data-dependent acquisition (DDA) strategy in positive mode with a precursor m/z scan range of 375–1,800 (resolution 140,000) triggering fragmentation and MS of the 12 most abundant ions per cycle (m/z scan range 200–2,000, resolution 17,500) and operating with a 15 s dynamic exclusion. Fragmentation was limited to ion charge states of +2 to +5. Spectra were assigned and LFQ intensities calculated across three biological replicates per condition using MaxQuant (45) Version 1.6.1.0 searching against the reviewed human proteome (UniProt/SwissProt accessed April 2016), considering fixed modification carbamidomethyl cysteine, variable modifications of N-terminal acetylation, and methionine oxidation, as well as up to two missed trypsin cleavages. Protein ratios between samples were calculated based on at least two common peptides, including unique and razor peptides. Protein identifications were filtered of decoys, contaminants, and proteins only identified by a modification site. Log2 transformed LFQ intensities for proteins assigned LFQ values in at least three samples were compared between conditions using Perseus Version 1.6.6.0 by a two-sided t-test (46). Missing values were imputed from a normal distribution (default settings). The mass spectrometry proteomics data have been deposited to the ProteomeXchange Consortium *via* the PRIDE (44) partner repository with the dataset identifier PXD024331.

### Abacavir-Specific T Cell Culture

To generate abacavir responsive T cells, 2–4 × 10^6^ HLA-B*57:01^+^ responder PBMCs were resuspended in 1 ml RF10 and placed in a single well of a 24 well tissue culture plate (Greiner Bio-One International AG, Austria). Autologous stimulator HLA-B*57:01^+^ PBMCs were incubated for 4–5 h at 37°C, 5% CO_2_ with 35 μM abacavir in 200 µl RF10. Stimulator PBMCs were subsequently added to responder PBMC at a 2:1 responder:stimulator ratio and the final volume of culture made up to 2 ml with RF10. Cells were incubated at 37°C, 5% CO_2_ and fed with fresh media as required to sustain cell outgrowth. On day 5 of culture, cell media were supplemented with rhIL-2 (Cetus) at 20 U/ml and maintained at this concentration thereafter. On days 11–14 cultures were tested for antigen specificity by restimulation with abacavir pulsed (incubated with 35 µM abacavir overnight, washed three times in RPMI 1640) C1R.B*57:01 followed by intracellular cytokine staining (ICS).

### Intracellular Cytokine Staining Assay

C1R.B*57:01 were incubated with 35 μM abacavir for set time periods (0–30 h) and HLA molecule export abrogated by 1% paraformaldehyde (PFA) fixation (ProSciTech, Australia) in PBS (2 h, room temperature). Cells were washed three times in RPMI 1640 to remove residual PFA, and stored overnight at 4°C in RH10 [RPMI 1640 (Life Technologies) supplemented with 10% human AB serum (HS; Sigma/Merck), 7.5 mM HEPES (MP Biomedicals), 100 U/ml Pen Strep (benzyl–penicillin/streptomycin, Life Technologies), 2 mM L-glutamine (MP Biomedicals), 76 μM *β*-mercaptoethanolamine (Sigma), and 150 μM non-essential amino acids (Life Technologies)]. C1R.B*57:01 were mixed with 1 × 10^5^ T cells at a 1:2 ratio in a volume of 200 μl fresh RH10. After 2 h at 37°C, 5% CO_2_, Brefeldin A (BFA; Sigma-Aldrich) was added to a final concentration of 10 μg/ml and incubated a further 4 h. Cells were stained with anti-CD4-PE [clone RPA-T4, Becton Dickinson (BD) Biosciences, USA] and anti-CD8-PerCP-Cy™5.5 (clone SK1, BD Biosciences, 25 min, 4°C), fixed with 1% PFA/PBS (20 min, room temperature), washed in PBS, then permeabilised with 0.3% saponin (Sigma-Aldrich) in PBS containing anti-Interferon-*γ* (IFN*γ*)-PE-Cy™7 (clone B27, BD Biosciences) and anti-tumour necrosis factor (TNF)-PE-V450 (clone Mab11, BD Biosciences), and acquired by flow cytometry using an LSR II flow cytometer and BD FACSDIVA™ software (BD). The percentage of CD8^+^ T cells producing IFN*γ* and TNF was determined *via* analysis using FlowJo software (BD).

### MHC I Pulse-Chase Maturation Assay

In order to assess the ability of abacavir to modulate HLA-B*57:01 maturation kinetics in various cell types, 60 µM abacavir was added to 2 × 10^7^ cells in 50 ml culture medium 20 h before the start of the assay, while 2 × 10^7^ cells were cultured in the absence of abacavir in otherwise identical conditions. Cells were harvested and incubated in 2 ml cysteine/methionine free RPMI (Sigma) containing 10% dialysed foetal bovine serum (Globepharm), 2 mM L-Glutamine (Sigma), and 60 µM abacavir, or no abacavir (as appropriate), for 40 min at 37°C. 3.8 MBq ^35^S EasyTag (Perkin Elmer) was added for 6 min. The labelling reaction was stopped by adding 20 ml RPMI containing 10% dialysed foetal bovine serum, 2 mM L-Glutamine, and 2 mM Methionine (Sigma). 4 ml samples were taken at time points, and cells were lysed in PBS containing 1% NP-40 (US Biological), 2 mM phenylmethylsulfonyl fluoride (Sigma), and 5 mM iodoacetamide (Sigma) and clarified by centrifugation at 16,060 *g* for 10 min. Supernatants were pre-cleared with protein A sepharose (Sigma) and incubated with W6/32 and Protein A sepharose beads for 1 h. MHC I molecules were eluted from the beads by heating at 95°C for 3 min in 1.5% SDS and 50 mM Tris-HCL pH 6.8. The sample was digested (or mock digested) with 500 U EndoH_f_ (NEB) using the manufacturer’s protocol. Proteins were separated by SDS PAGE, the gels dried, and bands were detected using Personal Molecular Imager FX (BIORAD) and quantified using Image J software. % of EndoH resistant material was calculated as a % of the total Endo H resistant and sensitive material.

### Pulse-Chase Thermostability Assay

In order to assess if abacavir stabilised HLA-B*57:01 complexes expressed by various cell types, 60 µM abacavir was added to 2 × 10^7^ cells in 50 ml culture medium 20 h before the start of the assay, while 2 × 10^7^ cells were cultured in the absence of abacavir in otherwise identical conditions. Cells were harvested and incubated in 2 ml cysteine/methionine free RPMI (Sigma) containing 10% dialysed foetal bovine serum (Globepharm), 2 mM L-Glutamine (Sigma), and 60 µM abacavir, or no abacavir (as appropriate), for 40 min at 37°C. 3.8 MBq ^35^S EasyTag (Perkin Elmer) was added for 6 min. The labelling reaction was stopped by adding 20 ml RPMI containing 10% dialysed foetal bovine serum, 2 mM L-Glutamine, and 2 mM Methionine (Sigma). 5 ml samples were taken at time points, and cells were lysed in PBS containing 1% NP-40 (US Biological), 2 mM phenylmethylsulfonyl fluoride (Sigma), and 5 mM iodoacetamide (Sigma) and clarified by centrifugation at 16,060 *g* for 10 min. Supernatants were pre-cleared with protein A sepharose (Sigma) for 1 h.

Each pre-cleared lysate was split into three tubes and incubated at 50, 37 or 4°C for 12 min then cooled on ice. MHC I molecules were precipitated with W6/32 and Protein A sepharose beads for 1 h. MHC I molecules were eluted from the beads by heating at 95°C for 3 min in 1.5% SDS, 50 mM Tris-HCl pH 6.8, 30% Glycerol, and 2 M 2-Mercaptoethanol. Proteins were separated by SDS PAGE. The gels were dried, and bands were detected using Personal Molecular Imager FX (BIORAD) and quantified using Image J software. Gels were re-hydrated and stained with Coomassie to quantify the IgG heavy chain. % of MHC I recovered after thermal denaturation was calculated by normalising MHC I band intensity against quantity of IgG heavy chain and represented as a proportion of material left compared to 4°C.

### Production of Nucleotides Encoding HLA B*57:01fos

DNA encoding the ER luminal domains of HLA-B*57:01 with C-terminal Fos leucine zipper sequence was created by three PCR reactions: nucleotides encoding the ER luminal domains of HLA-B*57:01 were amplified using primers 5′-AGCCATATGGGCTCCCACTCCATGAG-3′ and 5′-ACCGCCGGAACCTCCTGGCTCCCATCTCAG-3′ and HLA-B*57:01 DNA in pET30 plasmid; while nucleotides encoding the Fos leucine zipper were amplified from pET22b HLA-A*02:01fos [described in (47)] using primers 5′-CTGAGATGGGAGCCAGGAGGTTCCGGCGG-3′ and 5′-CGCAAGCTTTTAATGGGCGGCCAGGATGAACT-3′. The purified products from both PCR reactions were used in a third PCR reaction to create HLA-B*57:01fos using primers 5′-AGCCATATGGGCTCCCACTCCATGAG-3′ and 5′-CGCAAGCTTTTAATGGGCGGCCAGGATGAACT-3′. Following agarose gel electrophoresis and digestion of the purified product with restriction enzymes the sequence encoding HLA-B*57:01fos was cloned into pET22b (Novagen).

### Production of Monomeric Tapasin-Jun and Conjugated Tapasin-Jun-ERp57 C60A Proteins

Nucleotides encoding human tapasin-jun (47) were modified to allow purification *via* a TwinStrep affinity tag. First, nucleotides encoding a TEV protease cleavage site, TwinStrep affinity tag and stop codon were added to the pMT/BiP/V5-His A plasmid (ThermoFisher) as follows: a plasmid containing a synthetic gene encoding the TEV protease cleavage site, TwinStrep affinity tag and stop codon, flanked by nucleotides constituting 5′ XhoI and 3′ PmeI restriction enzyme sites (XhoI–TEV–TwinStrep–Stop–PmeI) was obtained from GeneArt (ThermoFisher). The nucleotides encoding the TEV–TwinStrep–Stop sequence were excised by digestion with XhoI and PmeI restriction enzymes and ligated into XhoI and PmeI digested pMT/BiP/V5-His A vector that had previously been modified to confer puromycin resistance as described in (47). Second, tapasin-jun was cloned into the pMT-BIP_TEV-TwinStrep (Puro) by one-step sequence and ligation independent cloning (48). Briefly, the vector pMT-BiP TEV-TwinStrep was digested with BglII and XhoI restriction enzymes while the sequence of tapasin-jun was amplified by PCR using the forward primer (5′- GGCCTCTCGCTCGGGAGATCTGGACCCGCGGTGATCG-3′) and reverse primer (5′- GAAATACAGGTTTTCCTCGAGGTTCATGACTTTCTGTTTAAG-3′). The digested vector and the PCR product were incubated at a molar ratio of 1:4 in the presence of T4 DNA polymerase (NEB) at room temperature for 2 min 30 s, then the reaction was left on ice for 10 min before competent bacteria were transformed.

DNA encoding ERp57, without the signal sequence, but with a C-terminal His_6_ tag located before the QEDL ER retrieval sequence was created by PCR using primers 5′-TAAAGATCTTCCGACGTGCTAGAACTCAC-3′ and 5′-GAAGAAGAAGAAGGCACATCACCATCACCATCACCAGGAGGATCTCTAAGAATTCCAC-3′ and human ERp57 DNA in pQE60 plasmid [described in (49)] and ligated to pMT-BiP plasmid modified to confer puromycin resistance following digestion of the purified product with restriction enzymes and agarose gel electrophoresis. The C60A mutation was introduced by PCR using primers 5′-GTGTGGACACGCCAAGAGACTTG-3′ and 5′-CAAGTCTCTTGGCGTGTCCACAC-3′ and ERp57-His_6_-QEDL in pMT-BiP (Puro).

S2 cells were transfected with either the pMT-BiP–Tapasin-Jun–TEV–TwinStrep (Puro) plasmid for the expression of monomeric tapasin-jun protein, or co-transfected with the pMT–BiP–Tapasin-Jun–TEV–TwinStrep (Puro) and pMT–BiP–ERp57 C60A His6 (Puro) plasmids for the expression of ERp57 C60A conjugated tapasin-jun proteins using Fugene6 transfection reagent (Promega) and OptiMEM medium (Fisher Scientific). Stable transfectants were generated by culturing the transfected cells in EX-CELL 420 Serum-Free Medium (Sigma) supplemented with 3 µg/ml puromycin (Melford).

Monomeric tapasin-jun or ERp57 C60A conjugated tapasin-jun proteins were purified from either cell supernatants or from cell pellets. Pooled cell supernatants were concentrated approximately five-fold using a 30 kDa membrane (Amicon). The pH of the concentrated supernatant was adjusted to pH 7–8 by the addition of binding buffer (50 mM Tris-HCl pH 8.0, 150 mM NaCl). BioLock biotin blocking solution (IBA Life Sciences) was added according to the manufacturer’s recommendations (to prevent free biotin present in the medium from binding irreversibly to the Strep-Tactin beads) and incubated at room temperature for 5 min, before being passed through a 0.22 µm filter. 10 ml of 50% Strep-Tactin Superflow High Capacity beads (IBA Life Sciences) was washed twice with 50 ml of binding buffer before being mixed with the filtered cell supernatant at 4°C overnight. Following centrifugation at 270 g for 2 min the beads were pooled and washed three times with 50 ml of binding buffer and transferred to a 10 ml column. 5 mM D-desthiobiotin diluted in binding buffer was used to elute the proteins with 1 ml fractions being collected. Protein containing fractions were pooled and concentrated by 30 kDa spin concentrator (ThermoFisher) and dialysed against 20 mM Tris-HCl pH 8.0, 150 mM NaCl at 4°C. The dialysed protein was recovered and concentrated further using a 2 ml 10 kDa spin concentrator. Cell pellets were resuspended in lysis buffer [100 mM Tris-HCl pH 8.0, 150 mM NaCl, 1% NP40, 10 mM MgCl2, Complete EDTA-free protease inhibitors (Roche), and 100 µl/g cell pellet of 2 mg/mL DNAse Sigma)], sonicated on ice, and mixed at 4°C for 30 min. The lysate was clarified by centrifugation at 17,000 *g*, 4°C. The supernatant was passed through a 0.22 µm filter and incubated with Strep-Tactin Superflow High Capacity beads as described for the purification of the proteins from the supernatants.

### Synthetic Peptides

For fluorescence polarisation experiments the following HLA-B*57:01 binding peptides were used: the UV-labile conditional peptide ligand LSSPVTKjF [j denotes 3-amino-3-(2-nitro)phenyl-propionic acid], the fluorescent peptides TSLK*SRVTI, LTTK*LTNTNI, ATFK*GIVRAI, NTVELRVK*I, KTFK*DVGNLL, KVFK*LQTSL, VTKK*TYEIW, ITTK*AISRW, RVDPAK*GLFYF (K* denotes TAMRA labelled lysine), non-labelled peptides TSLKSRVTI, LTTKLTNTNI, ATFKGIVRAI, NTVELRVKI, KTFKDVGNLL, KVFKLQTSL, VTKKTYEIW, and RVDPAKGLFYF. TAMRA labelled and unlabelled peptides were synthesised by GL Biochem Ltd (Shanghai, China). The UV labile peptide was synthesised by Peptide Protein Research Ltd (Fareham, UK). Unlabelled peptides used in LC-MRM-MS (**Supplementary Table 1**) were synthesised by GL Biochem Ltd (Shanghai, China).

### Production of Peptide-Loaded HLA-B*57:01fos Complexes

Peptide loaded HLA-B*57:01fos complexes were obtained as described (50) by refolding solubilised inclusion bodies of HLA-B*57:01fos heavy chains with human *β*_2_m and UV-labile HLA-B57-conditional peptide ligand.

### Fluorescence Polarisation Experiments

Fluorescence polarisation measurements were taken using a SpectraMax i3x Multi-Mode Microplate Reader (Molecular Devices) with rhodamine detection cartridge. All experiments were conducted at 25°C and used PBS supplemented with 0.5 mg/ml bovine-gamma-globulin (Sigma), and a 20 fold molar excess of human *β*_2_m (Fitzgerald) in a volume of 60 µl. Binding of TAMRA-labelled peptides is reported in millipolarisation units (mP) and is obtained from the equation mP = 1,000 × (S – G × P)/(S + G × P), where S and P are background subtracted fluorescence count rates (S = polarisation emission filter is parallel to the excitation filter; P = polarisation emission filter is perpendicular to the excitation filter) and G (grating) is an instrument and assay dependent factor.

### Association Rate Measurements

Peptide-receptive HLA-B*57:01fos were obtained by exposing HLA-B*57:01fos complexes loaded with UV labile conditional peptide ligand to ~360 nm light for 20 min at 4°C (“UV exposed” hereafter). The binding of fluorescent peptides to UV exposed HLA-B*57:01fos was monitored in the absence or presence of excess (60 µM) abacavir or tapasin-jun or tapasin-jun ERp57 C60A conjugate. Comparable results were obtained with either monomeric tapasin-jun or ERp57 C60A conjugated tapasin-jun proteins. Most experiments used tapasin-jun proteins at a concentration of 0.254 µM, but some experiments used different preparations of tapasin-jun protein, which required higher concentrations (up to 0.75 µM) to achieve comparable function. Most experiments used 0.55 µM HLA-B*57:01fos molecules, but a minority of experiments involved different preparations of HLA-B*57:01fos which required higher concentrations of HLA-B*57:01fos, up to a maximum of 3.18 µM HLA-B*57:01fos, to achieve equivalent polarisation levels. Comparable results were obtained. Peptide concentrations varied between experiments, but gave comparable results: KTFK*DVGNLL 0.015–0.045 µM, TSLKSRVTI 0.015–0.083 µM, KVFK*LQTSL 0.015–0.1 µM, ATFK*GIVRAI 0.015–0.092 µM, NTVELRVK*I 0.015–0.210 µM, VTKK*TYEIW 0.015–0.106 µM, RVDPAK*GLFYF 0.015–0.087 µM, ITTK*AISRW 0.015–0.353 µM, LTTK*LTNTNI 0.006–0.024 µM.

### Dissociation Rate Measurements

HLA-B*57:01fos molecules were UV exposed and then allowed to bind fluorescent peptides overnight at room temperature. Florescence polarisation measurements were taken after the addition of excess non-labelled competitor peptide (otherwise identical to the labelled peptide) in the absence or presence of 60 µM abacavir or conjugated tapasin-jun-ERp57 C60A proteins. Most experiments used tapasin-jun proteins at a concentration of 0.254 µM, but some experiments used different preparations of tapasin-jun protein, which required higher concentrations (up to 0.949 µM) to achieve comparable function. Most experiments used 0.55 µM HLA-B*57:01fos molecules, but some experiments involved different preparations of HLA-B*57:01fos which required higher concentrations of HLA-B*57:01fos, up to a maximum of 1.38 µM HLA-B*57:01fos, to achieve equivalent polarisation levels. Comparable results were obtained. Peptide concentrations varied between experiments, but gave comparable results: KTFK*DVGNLL 0.015–0.045 µM, TSLKSRVTI 0.015–0.034 µM, KVFK*LQTSL 0.015–0.039 µM, ATFK*GIVRAI 0.015–0.063 µM, NTVELRVK*I 0.015–0.165 µM, VTKK*TYEIW 0.015–0.028 µM, RVDPAK*GLFYF 0.015–0.082 µM, ITTK*AISRW 0.015 µM.

## Results

### Abacavir Induced Changes in the HLA-B*57:01 Immunopeptidome Increase Over Time

We previously characterised the impact of abacavir exposure on the immunopeptidome of HLA-B*57:01 molecules isolated from C1R.B*57:01 cells and determined the sequences of novel drug induced ligands by liquid chromatography-tandem mass spectrometry (LC-MS/MS) (25). This workflow enabled identification of peptides without prior knowledge of sequence but is biased towards those of highest intensity during LC-MS/MS. In contrast, liquid chromatography-multiple reaction monitoring–mass spectrometry (LC-MRM–MS) specifically targets known peptides based on knowledge of their precursor ion mass and sequence specific fragmentation patterns. LC-MRM–MS strategies have been shown to facilitate the detection of both high and low abundance peptides against the complex background of the immunopeptidome and facilitate relative peptide quantitation (42, 51–53). Therefore, to follow the perturbation of the immunopeptidome over time, we designed a LC-MRM–MS based approach to track changes in the relative abundance of a subset of HLA-B*57:01 ligands within the immunopeptidome. Based on this strategy, 58 HLA-B*57:01 ligands were robustly detected; 35 that were originally identified in both treated and untreated cells, 22 that were identified only in treated cells, and one that was previously identified in untreated cells alone (see **Supplementary Table 1** for details). These peptides possessed predominantly canonical HLA-B*57:01 anchor residues at P2 of the peptide (Ser, Thr, Ala, or Val), whilst P*Ω* residues included both the preferred canonical aromatic P*Ω* anchors (Trp, Phe, Tyr) and aliphatic residues that increase in representation following exposure to abacavir (Ile, Leu and Val). As C1R.B*57:01 also expresses the HLA class I molecule HLA-C*04:01, five peptides identified as ligands of HLA-C*04:01 in parental C1R cells (54) were also targeted for detection as an internal drug-specificity control.

**Figure 1:**
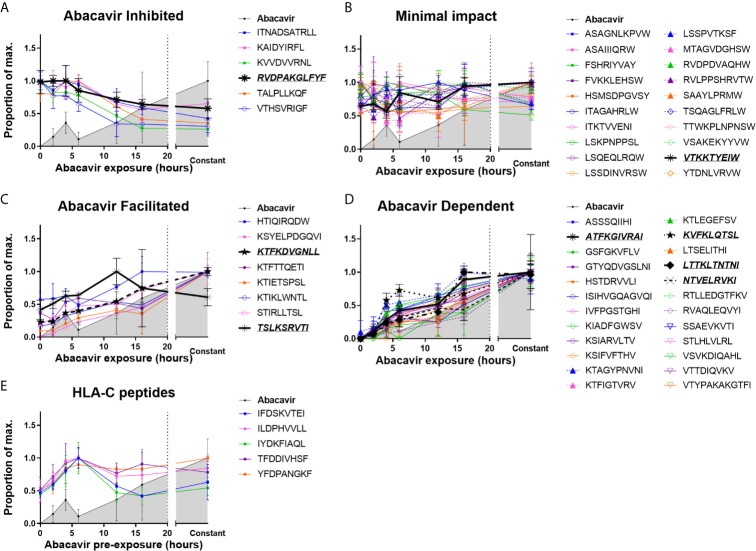
Abacavir perturbs the HLA-B*57:01 immunopeptidome by increasing or decreasing the contribution of distinct subsets of peptides. Analysis of 58 selected HLA-B*57:01 peptide ligands, isolated from 10^8^ C1R.B*57:01 cells after 0–16 h, or constant, abacavir treatment using LC-MRM–MS demonstrated four main patterns of impact on contribution to the immunopeptidome; **(A)** inhibition, **(B)** minimal impact, **(C)** facilitated (presented by HLA-B*57:01 of untreated cells but increased in abundance during abacavir treatment) and **(D)** dependent (only presented by HLA-B*57:01 of cells exposed to abacavir). Perturbation of HLA-B*57:01 ligands increased with time, coincident with increased co-purification of abacavir. Perturbations were not mirrored by the HLA-C ligands analysed **(E)**. Peptide and abacavir abundances are shown as a proportion of the maximum normalised peak area detected and are portrayed as the mean of the three biological replicates (mean +/− SD). The decreased recovery of abacavir at the 6 h point is likely to reflect an experimental artefact in the off line fractionation prior to the LC-MS. Importantly there was not a similar decrease in the amount of peptide material eluted at this time point. Peptides that were chosen for *in vitro* peptide binding and dissociation experiments are in italics and underlined.

MHC I molecules were isolated from C1R.B*57:01 cells that had been cultured for 0–16 h, or continuously, in the presence of abacavir. Changes in the immunopeptidome were evident after 2 h of abacavir treatment and became more prominent during the 16 h time course of the experiment. This coincided with the co-purification of increasing amounts of HLA-B*57:01-bound abacavir (**Figure 1** and **Supplementary Figure 1**). The changes in the abundance of peptides in response to abacavir exposure could be categorised into four groups. One group, consisting of six peptides, was abacavir inhibited (decreased in abundance over exposure time, **Figure 1A**). Another group of 20 peptides experienced minimal impact (no marked change in abundance, **Figure 1B**). Eight peptides were observed to be abacavir facilitated (present in the absence of abacavir but increased in abundance with abacavir exposure, **Figure 1C**), and the remaining 24 peptides were abacavir dependent (not robustly detected in the absence of abacavir and increased in abundance during abacavir exposure, **Figure 1D**). Ligands of HLA-C are shown for comparison in **Figure 1E**.

#### Abacavir Independent Ligands

The majority of the 20 peptides that were minimally impacted by abacavir terminated in canonical P*Ω* anchor residues for HLA-B*57:01 (25, 55) (**Figure 1B** and **Supplementary Figure 1**, orange symbols and line): 15 peptides terminated in Trp, whilst a further three peptides terminated in less favoured canonical residues, Tyr and Phe. These included LSSPVTKSF for which crystal structures of this peptide in complex with HLA-B*57:01 demonstrate the occupation of the abacavir binding site by P7 Lys and P9 Phe residues (25, 26). Consistent with their conformity to the canonical HLA-B*57:01 peptide binding motif most peptides within this category were predicted to be strong binders of HLA-B*57:01 by NetMHC4.0 (56, 57) (**Supplementary Figure 2**), suggesting these peptides are high affinity ligands, which are not markedly impacted by competition from abacavir and abacavir-induced peptide ligands. The two remaining peptides, ITKTVVENI and LSKPNPPSL, were predicted to bind B*57:01 with low affinity (**Supplementary Figure 2**, red and green symbols) and, despite their P*Ω* residues being considered conducive to binding in the presence of abacavir, these peptides appear to be well represented in the constitutive HLA-B57 immunopeptidome.

#### Abacavir Inhibited Peptides

We observed that six of the peptides in our panel decreased in abundance following the addition of abacavir (**Figure 1A** and **Supplementary Figure 1**, red symbols and line). Three peptides terminated in the canonical Phe residue at P*Ω*, whilst the remaining three peptides terminated in the combination of Leu at P*Ω* and Arg at P*Ω*-2. We have previously observed that Phe at P*Ω* provides less stability to HLA-B*57:01 complexes than Trp (55) and indeed, two of these peptides were predicted to bind B*57:01 with low affinity (**Supplementary Figure 2** and **Supplementary Table 1**). This suggests that these ligands may be displaced from the repertoire in preference of higher stability HLA-B*57:01-abacavir–peptide complexes. Similarly, whilst Arg at P*Ω*-2 can act as a secondary anchor for HLA-B*57:01, forming a salt-bridge with Asp114 of the E-pocket and stabilising the peptide within the groove (55), the three peptides with the combination of Leu at P*Ω* and Arg at P*Ω*-2 were also predicted to bind with low affinity (**Supplementary Figure 2** and **Supplementary Table 1**). This suggests that these peptides are vulnerable to dissociation due to the interaction between Arg at P*Ω*-2 and Asp114 being out-competed by the presence of abacavir within the antigen-binding cleft.

#### Abacavir Enhanced Peptides

We found eight peptides that were detected at relatively low abundance in the immunopeptidome of untreated cells but substantially increased when the cells were grown in abacavir supplemented media (**Figure 1C** and **Supplementary Figure 1**, yellow symbols and line). Seven of these peptides terminated in aliphatic residues (three Ile and four Leu), whilst the remaining peptide, HTIQIRQDW terminated in the preferred canonical Trp residue. Of the eight peptides, HTIQIRQDW possesses the highest predicted binding affinity for HLA-B*57:01 (**Supplementary Figure 2**, blue symbol). Given C-terminal Trp is likely to clash with abacavir binding, the modest enhancement of the HTIQIRQDW peptide may indicate that canonical peptides such as HTIQIRQDW benefit from the altered competition between peptides that follows the addition of abacavir.

Presentation of another 24 peptides appeared entirely dependent on abacavir (**Figure 1D** and **Supplementary Figure 1**, green symbols and line). All of these peptides terminated in aliphatic residues considered characteristic of abacavir-induced peptide ligands: 13 peptides terminated in Ile, three in Leu and eight in Val, whilst only ATFKGIVRAI possessed Arg at P*Ω*-2. The absence of abacavir dependent peptides from the constitutive immunopeptidome strongly suggests that abacavir is obligatory for these peptides to bind to HLA-B*57:01. Indeed, most peptides within this category were predicted non-binders of HLA-B*57:01 (**Supplementary Figure 2**).

Of note, the abacavir facilitated peptide KTIKLWNTL is derived from Receptor of activated protein C kinase 1 (RACK1), the same source protein as YTDNLVRVW, which was minimally impacted by abacavir. Similarly, the abacavir dependent peptide LTSELITHI and abacavir inhibited peptide KVVDVVRNL are derived from Interferon-induced GTP-binding protein Mx1 (MX1). Thus, it is unlikely that differences in presentation kinetics between these peptide pairs are the result of changes in source protein abundance.

### Abacavir Has Minimal Impact on the C1R.B*57:01 Proteome

**Figure 2:**
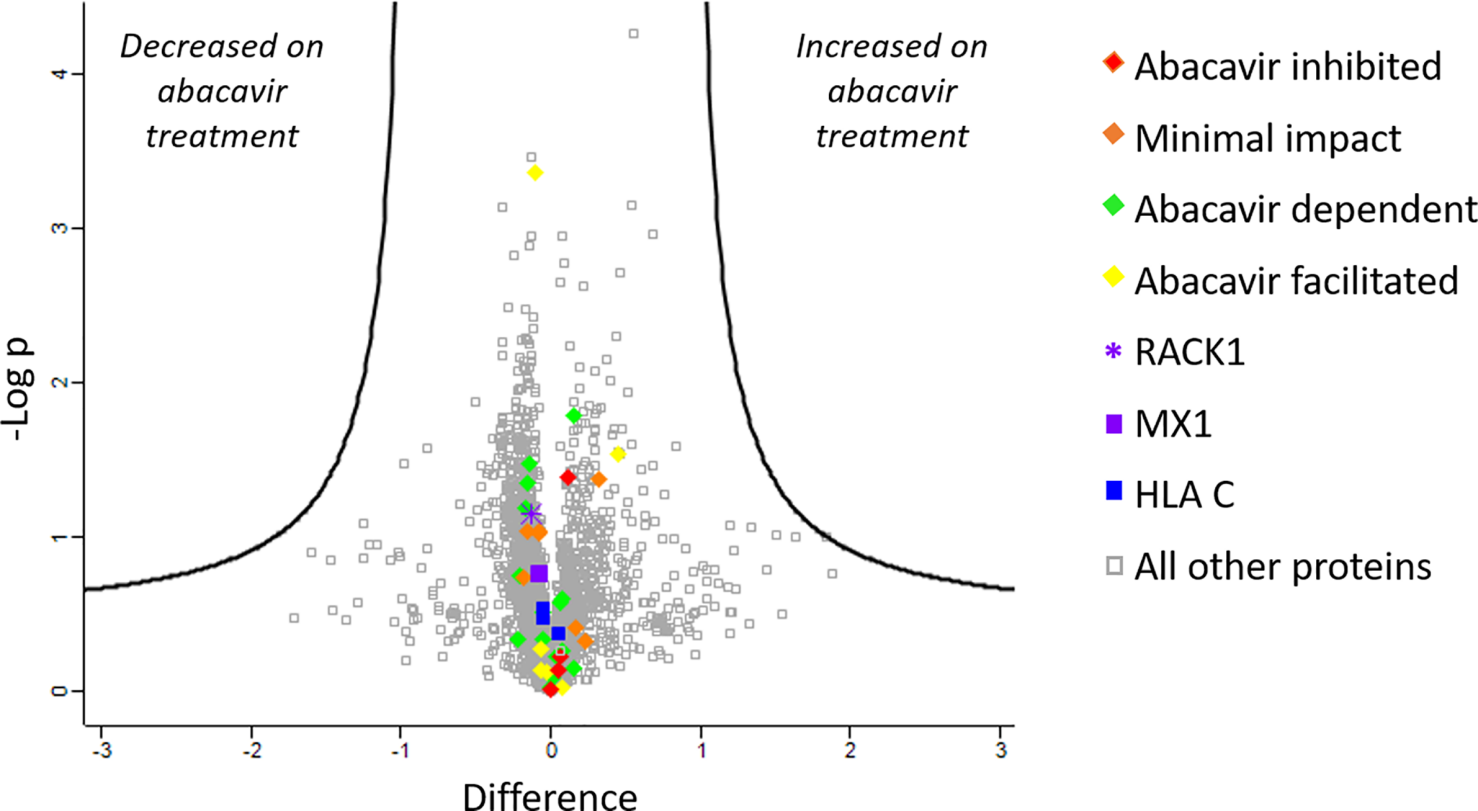
Abacavir does not perturb the proteome of C1R.B*57:01. Comparison of protein abundance between untreated and abacavir treated (48 h, 35 µM) C1R.B*57:01 cells (three replicates per treatment) revealed no major perturbation of the cellular proteome. The volcano plot depicts the difference (log_2_FC) *vs* -Logp of 2,306 proteins between untreated and treated conditions. A 5% FDR and a slope of 1 were used as the cut-off for significance (thick black line). No proteins were significantly perturbed by abacavir treatment; furthermore identified source proteins for the HLA ligands analysed clustered close to 0 on the difference axis. Source proteins for HLA ligands from the different subsets analysed are shown as indicated in the key. The purple asterisk shows RACK1, the source protein of peptides KTIKLWNTL (abacavir facilitated), and YTDNLVRVW (minimal impact), and the purple square shows MX1, the source of peptides LTSELITHI (abacavir dependent) and KVVDVVRNL (abacavir inhibited). All other proteins are shown by grey squares.

To further confirm that changes in the abundance of HLA-bound peptides were not due to significant changes in source protein abundance induced by abacavir treatment, we analysed the cellular proteome of C1R.B*57:01 cells that were cultured in the presence or absence of abacavir for 48 h. Cells were lysed before the cellular proteins were isolated and digested and subjected to LC-MS/MS and label free quantitation (LFQ) analysis. A total of 3,077 proteins were identified, of which 2,306 were robustly detected in at least three samples and used for LFQ. This included the source proteins for 37 of the 58 HLA-B*57:01 ligands and three of the five HLA-C*04:01 ligands incorporated in the immunopeptidome analysis. Importantly, no protein showed significant changes in expression following abacavir treatment (**Figure 2**).

### Immunogenicity of Abacavir Treated Cells Increases Over Time

**Figure 3:**
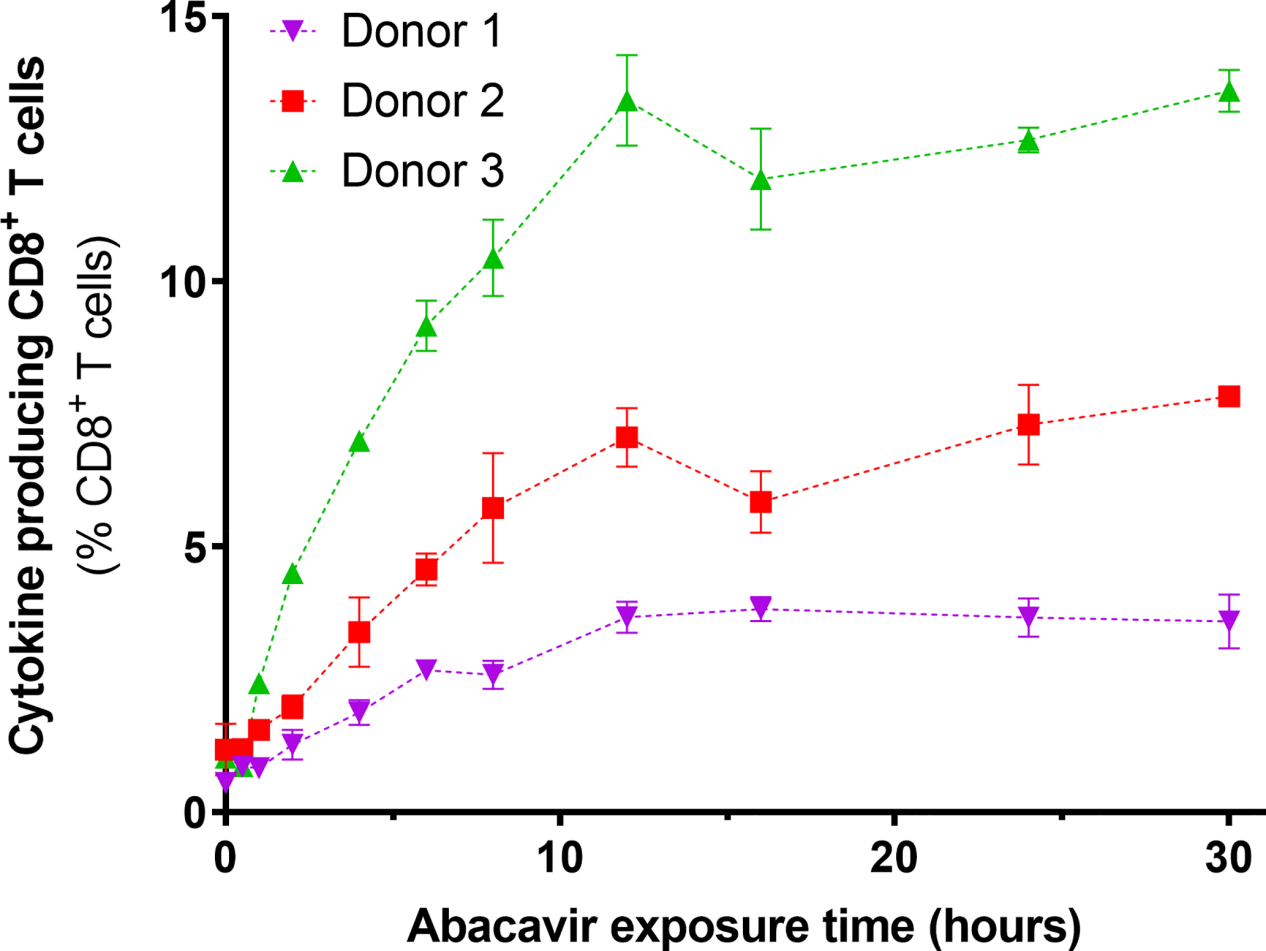
Immunogenicity of HLA-B*57:01+ antigen presenting cells increases with abacavir exposure time. C1R.B*57:01 cells were cultured in 35 µM abacavir for 0–30 h prior to fixation in paraformaldehyde. Immunogenicity was then gauged by their ability to stimulate cytokine (IFNγ and/or TNF) production in CD8^+^ abacavir responsive T cells during a 6 h stimulation assay, detected *via* intracellular cytokine staining. Responses from T cell lines derived from three different HLA-B*57:01^+^ donors are shown. Donor 1 T cells were assayed in triplicate, whilst donor 2 and 3 T cells were assayed in duplicate in a separate experiment (mean +/− SD).

To determine if the observed changes in the immunopeptidome occurred over a similar time scale to the acquisition of immunogenicity, C1R.B*57:01 cells were exposed to abacavir for various lengths of time, fixed and assayed for their ability to elicit cytokine production (IFN*γ* and/or TNF) from abacavir responsive CD8^+^ T cell lines. For the three T cell lines tested, an increase in antigen presenting cell immunogenicity was observed over the first 12 h of abacavir exposure, before levelling out (**Figure 3**).

### Intracellular Biogenesis of Stable HLA-B*57:01–Peptide Complexes Is Unchanged by Abacavir Treatment

**Figure 4:**
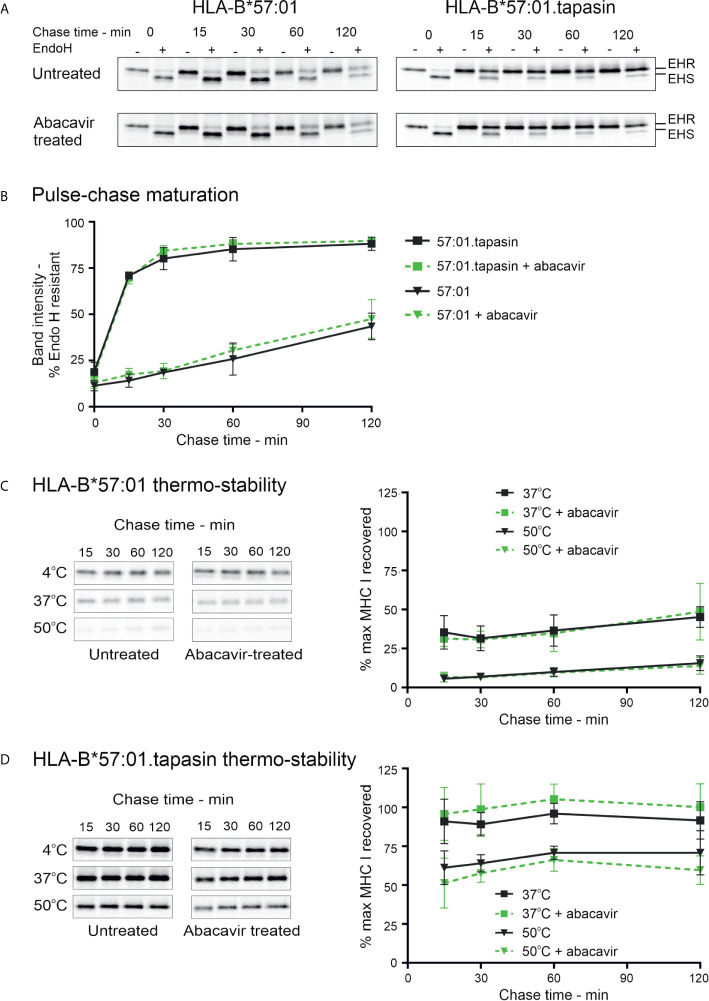
Abacavir does not significantly alter maturation or global stability of HLA-B*57:01. **(A)** and **(B)** Maturation [acquisition of resistance to digestion with endoglycosidase-H (Endo-H)] of HLA-B*57:01 molecules was determined by pulse chase in 721.220.B*57:01 or 721.220.B*57:01.tapasin cells, immunoprecipitation of MHC I molecules, and digestion with endoglycosidase-H. **(A)** depicts representative SDS-PAGE gels for untreated and abacavir treated cells; only the HLA-B*57:01 heavy chain bands are shown. The location of Endo-H resistant (EHR) and Endo-H sensitive (EHS) bands is distinguished by their altered migration pattern. **(B)** The % EHR material was calculated [EHR/(EHR+EHS)] and plotted as the mean (+/− SD) of three or four replicate experiments for abacavir treated and untreated cells respectively excluding time point 15, which was only included in two experiments. Abacavir had no apparent impact on HLA-B*57:01 maturation in either the presence or absence of tapasin. **(C)** and **(D)** Pulse-chase thermostability assays were performed in 721.220.B*57:01 **(C)** and 721.220.B*57:01.tapasin **(D)** cells: lysates were prepared from pulse radio-labelled cells and incubated at either 4, 37, or 50°C before *β*_2_m associated MHC I molecules were immunoprecipitated using W6/32 antibody and resolved by SDS-PAGE. Representative SDS-PAGE gels for untreated and abacavir treated cells depict the HLA-B*57:01 heavy chain bands recovered at each chase time after thermal denaturation. Graphs show the amount of HLA-B*57:01 molecules recovered after thermal denaturation at 37 and 50°C as a percentage of that recovered at 4°C (mean +/− SD of four or five replicate experiments for abacavir treated and untreated cells respectively).

Polymorphic residues of MHC I allotypes that interact with the C-terminal region of the peptide, including residues 114 and 116, are implicated in determining the dependence upon tapasin for the selection and assembly with high affinity peptides (2, 3, 58). HLA-B*57:01 has been classified as tapasin-dependent because it is poorly expressed at the cell surface in the absence of tapasin (28) (**Supplementary Figure 3**). Given that abacavir binds beneath the C-terminus of the peptide and interacts with residues that influence tapasin dependence (23, 25), we sought to determine whether abacavir i) promotes HLA-B*57:01 maturation independently of, or in conjunction with, tapasin, and/or ii) alters the dependence of HLA-B*57:01 upon tapasin for optimal peptide selection (26, 28). The tapasin-deficient 721.220 cell line (27) and the tapasin reconstituted 721.220.tapasin cell lines have previously been used to measure the rate and extent of intracellular peptide selection by newly synthesised MHC I molecules using pulse chase experiments (3). We therefore utilised this system to explore the maturation and stability of newly generated HLA-B*57:01 complexes in the presence or absence of abacavir. Pulse-chase maturation assays showed impaired acquisition of resistance to digestion with Endoglycosidase-H (Endo-H) by HLA-B*57:01 molecules in the absence of tapasin. Only ~25% of labelled complexes gained Endo-H resistance over the first 60 min of the assay compared to ~85% of HLA-B*57:01 complexes in tapasin expressing cells. This proportion of Endo-H resistant complexes increased to ~44% after 2 h (**Figures 4A, B**). Abacavir did not significantly alter this pattern of biogenesis. The maturation of HLA-B*57:01 molecules was also unaffected by abacavir in tapasin-reconstituted cells (**Figures 4A, B**).

In agreement with the lack of effect on HLA-B*57:01 maturation in tapasin deficient 721.220 cells (**Figures 4A, B**), abacavir had no significant impact on the thermal stability profile of HLA-B*57:01 in the absence of tapasin (**Figure 4C**). As anticipated, tapasin reconstituted cells produced more thermostable complexes (**Figure 4D**). This suggests that abacavir does not compensate for the absence of tapasin to promote the formation of stable HLA-B*57:01 complexes. These data were consistent with measurements of cell-surface expression and stability of HLA-B*57:01 molecules which showed that whilst tapasin increased surface expression as observed previously (28) and prolonged the half-life of HLA-B*57:01 molecules at the cell surface, abacavir did not significantly alter either the expression level or the stability of cell surface HLA-B*57:01 molecules (**Supplementary Figure 3**). This suggests that tapasin, but not abacavir, can increase the proportion of high stability HLA-B*57:01 complexes generated. Collectively, these data confirm that tapasin improves the ability of HLA-B*57:01 molecules to select high quality (ligands that generate complexes stable at 50°C) or medium quality (ligands that generate complexes stable at 37°C but not 50°C) peptide cargo.

### Abacavir Enhances the Loading of Specific Peptides From the Immunopeptidome of HLA-B*57:01

**Figure 5:**
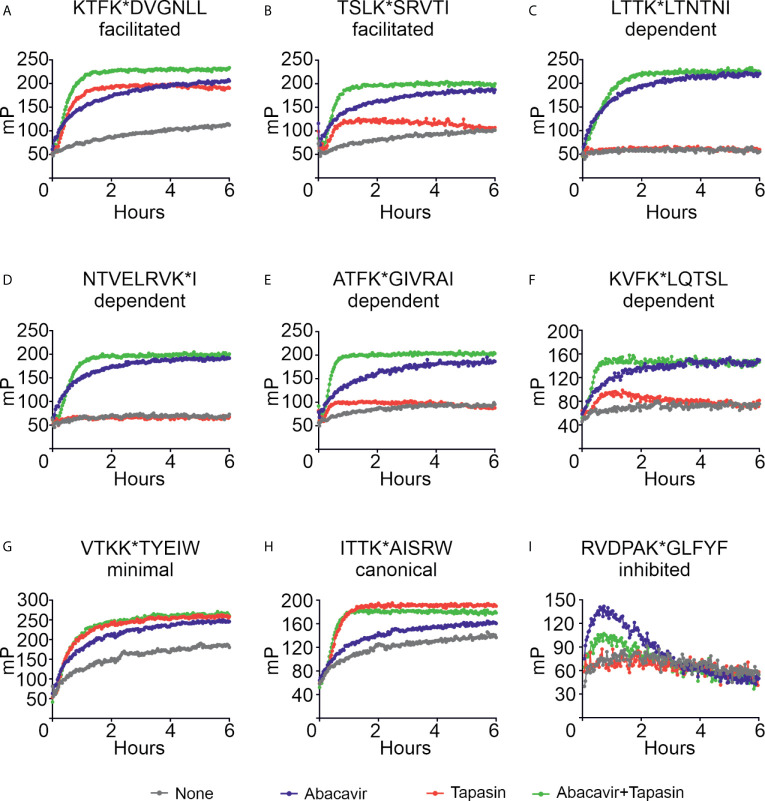
Abacavir diversifies the HLA-B*57:01 peptide repertoire by enhancing the loading of specific peptides. Conditional ligand loaded HLA-B*57:01fos molecules were UV exposed before the binding of the indicated peptide (**A** = KTFK*DVGNLL, **B** = TSLK*SRVTI, **C** = LTTK*LTNTNI, **D** = NTVELRVK*I, **E** = ATFK*GIVRAI, **F** = KVFK*LQTSL, **G** = VTKK*TYEIW, **H** = ITTK*AISRW, **I** = RVDPAK*GLFYF) was followed at 25°C in the presence or absence of excess abacavir and/or monomeric or ERp57 C60A conjugated tapasin-jun. Binding of fluorescent peptide is reported in millipolarisation units (mP). Unbound fluorescent peptide is assumed to have an mP level of 50. Data shown are representative of triplicate or greater experiments. The extent that tapasin, abacavir, or the combination of both enhanced the binding of fluorescent peptides to HLA-B*57:01fos molecules in the replicate experiments is presented in **Figure 6**. Colour coding is as follows: grey—peptide alone, blue—abacavir, red—tapasin, green—abacavir + tapasin.

We next investigated the mechanism by which abacavir changes the composition of the HLA-B*57:01 immunopeptidome by conducting *in vitro* peptide binding experiments. We first measured the binding of a panel of fluorescently labelled peptides to HLA-B*57:01 in the absence or presence of abacavir, or the MHC I co-factor tapasin or both. This peptide panel included two peptides, KTFK*DVGNLL and TSLK*SRVTI (KTF and TSL hereafter), classified by quantitative mass spectrometry as being abacavir facilitated. Consistent with this observation, HLA-B*57:01fos molecules displayed a limited intrinsic ability to bind either KTF or TSL peptides (**Figures 5A, B**, grey lines), with binding strongly enhanced when either abacavir (blue lines) or tapasin (red lines) was included. When tapasin and abacavir were both added (green lines), binding of both peptides occurred faster than in the presence of tapasin or abacavir alone.

Four peptides within our panel were classified by quantitative mass spectrometry as being dependent on abacavir. There was negligible to very little intrinsic binding of any of these peptides to the B*57:01fos molecules (**Figures 5C–F**, grey lines). Abacavir strongly enhanced binding of all four peptides (blue lines) although enhancement of the binding of the ATF peptide was less pronounced than the other three abacavir dependent peptides, consistent with Arg at P*Ω*-2 being unfavourable for the binding of abacavir. Tapasin did not affect the binding of the LTT or NTV peptides, and only slightly enhanced binding of the ATF and KVF peptides during the first few hours of the experiment (**Figures 5C–F**, red lines). Notably the binding of all four peptides occurred faster with the combined addition of abacavir and tapasin in comparison to that which was evident in the presence of abacavir or tapasin alone (**Figures 5C–F**, blue, red and green lines).

Our panel also included two canonical HLA-B*57:01 peptides VTKK*TYEIW and ITTK*AISRW (VTK and ITT hereafter) with VTK classified by quantitative mass spectrometry as being minimally affected by abacavir. Both peptides bound to HLA-B*57:01 molecules (**Figures 5G–H**, grey lines), but in contrast to the abacavir dependent peptides, tapasin (red lines) catalysed binding of the VTK and ITT peptides to a greater extent than abacavir (blue lines). The combined addition of tapasin and abacavir did not enhance the binding of the VTK and ITT peptides beyond the enhancement that tapasin afforded (green lines).

The last peptide included in our panel was RVDPAK*GLFYF (RVD hereafter), which quantitative mass spectrometry experiments indicated decreased in the immunopeptidome when cells were cultured in abacavir. Consistent with this peptide being predicted to bind B*57:01 with very low affinity (**Supplementary Figure 2**, blue symbol), we observed poor binding of RVD to B*57:01 molecules (**Figure 5I**, grey line). Surprisingly, binding of RVD was enhanced by abacavir during the first hour of the experiment when tapasin was absent (blue line); but tapasin partially mitigated this effect (green line), providing an example of how some peptides may be “edited out” of the repertoire.

**Figure 6:**
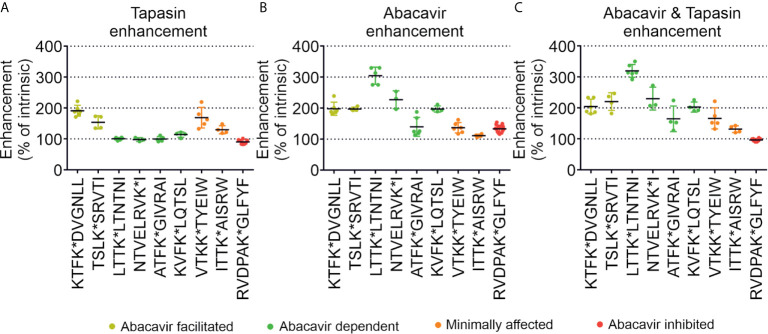
Tapasin and abacavir differentially enhance the binding of fluorescent peptides. The extent that **(A)** tapasin, **(B)** abacavir, or **(C)** the combination of both enhanced the binding of fluorescent peptides to HLA-B*57:01fos molecules was compared over independent experiments. For each polarisation measurement taken in an experiment the enhancement factor was calculated by: dividing polarisation in the presence of tapasin (or abacavir or both) by the intrinsic polarisation level; multiplying by 100 to obtain a percentage; and calculating the mean enhancement for the experiment. The enhancement factors obtained from different experiments are shown as dots, with the mean average shown as a horizontal black bar, and the standard deviation of multiple experiments shown as a coloured vertical line edged with horizontal bars. Peptides are coloured according to their classification by mass spectrometry: abacavir facilitated = yellow, abacavir dependent = green, minimally affected = orange, abacavir inhibited = red.

To enable comparison of the effects of abacavir, tapasin, or the combination of abacavir and tapasin on the binding of our panel of peptides, we calculated how much each enhanced the binding of specific peptides (**Figure 6**). **Figure 6A** shows that tapasin enhanced the binding of abacavir-facilitated peptides (KTF and TSL) to a greater extent than abacavir dependent peptides, consistent with peptides belonging to the latter category being absent from, or poorly represented in, the immunopeptidome of untreated cells. **Figure 6B** shows that binding of abacavir-facilitated (KTF and TSL) and abacavir-dependent (LTT, NTV and KVF) peptides increased to a greater extent than those peptides that were minimally affected (VTK and ITT) or decreased in representation within the immunopeptidome (RVD) following the addition of abacavir. The combined addition of tapasin and abacavir produced a similar enhancement hierarchy to that observed for abacavir (**Figure 6C**).

### Abacavir Diversifies the HLA-B*57:01 Peptide Repertoire by Decreasing Dissociation of Specific Peptides

**Figure 7:**
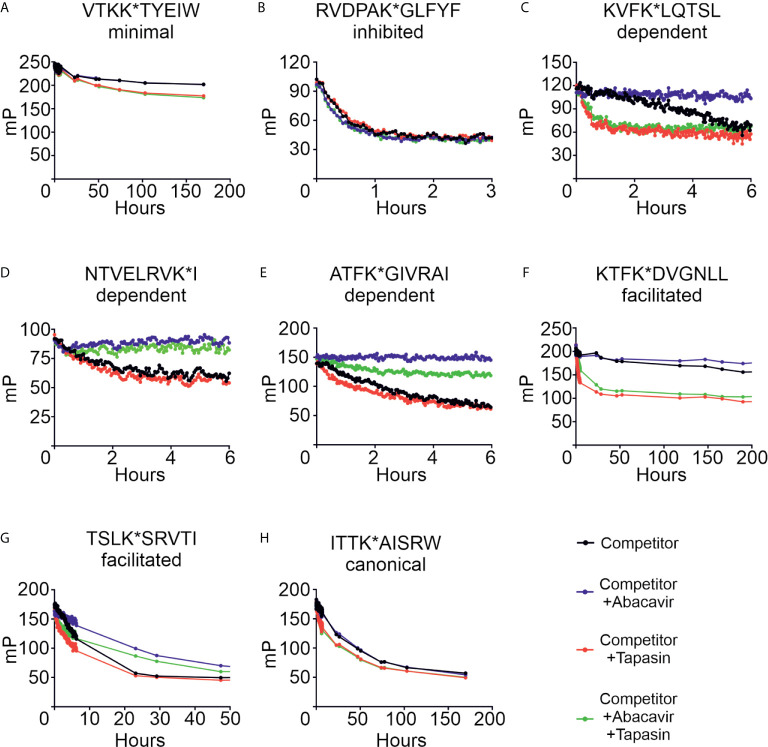
Abacavir diversifies the HLA-B*57:01 peptide repertoire by decreasing dissociation of specific peptides. Conditional ligand loaded HLA-B*57:01fos molecules were UV exposed and incubated with the indicated fluorescent peptide (**A** = VTKK*TYEIW, **B** = RVDPAK*GLFYF, **C** = KVFK*LQTSL, **D** = NTVELRVK*I, **E** = ATFK*GIVRAI, **F** = KTFK*DVGNLL, **G** = TSLK*SRVTI, **H** = ITTK*AISRW). Fluorescence polarisation measurements were taken after the addition of excess unlabelled competing peptide in the absence or presence of abacavir and/or conjugated tapasin-jun-ERp57 C60A proteins. Data shown are representative of triplicate or greater experiments.

We next characterised the effect that abacavir has on peptide dissociation. Conditional ligand loaded HLA-B*57:01fos molecules were UV exposed and then incubated with eight of the labelled peptides from our panel before unlabelled competing peptide, used to block rebinding of the labelled peptide, was added in the presence or absence of abacavir, tapasin, or both. Dissociation of the labelled peptides was measured by fluorescence polarisation. **Figure 7** and **Supplementary Table 2** show that the peptides dissociated from HLA-B*57:01fos molecules at different rates (black lines), ranging from hundreds of hours (*e.g.* VTK, **Figure 7A**) to minutes (*e.g.* RVD, **Figure 7B**). Notably, the fast off-rate of RVD is consistent with the depletion of this peptide from the immunopeptidome upon exposure to abacavir (**Figure 1A**), whilst the slow off-rate of VTK may explain the persistence of this peptide in the immunopeptidome (**Figure 1B**). Tapasin has been shown to enhance the dissociation of peptides from MHC molecules, the extent of this effect being peptide-dependent (31). In accordance, we saw a range of tapasin dependent enhancements of dissociation (red lines), ranging from significant (*e.g.* KTF, **Figure 7F**) to minimal (*e.g.* NTV, **Figure 7D**).

The addition of abacavir together with competing peptide (blue lines) led to a pronounced decrease in the dissociation of the abacavir dependent (KVF, NTV and ATF, **Figures 7C–E**) and abacavir facilitated (KTF and TSL, **Figures 7F–G**) peptides. In contrast, the addition of abacavir and competing peptide had minimal impact on the dissociation of the VTK and ITT peptides (**Figures 7A, H**), consistent with minimal impact of abacavir on the contribution of VTK to the immunopeptidome. Notably, we observed that the fast dissociation rate of the RVD peptide from B*57:01fos molecules was modestly enhanced by the addition of abacavir and competing peptide (**Figure 7B**), providing insight into why this peptide is depleted from the immunopeptidome following exposure to abacavir. In summary, the combined addition of competing peptide, abacavir and tapasin (green lines) affected the dissociation of the assayed peptides in different ways, which is likely to reflect the variation in the enhancement of peptide dissociation that tapasin mediates (*e.g.* significant for the KVF and KTF peptides, but minimal for the NTV peptide) combined with the variation in the stabilisation that the drug affords to specific HLA-B*57:01–abacavir–peptide complexes.

## Discussion

**Figure 8:**
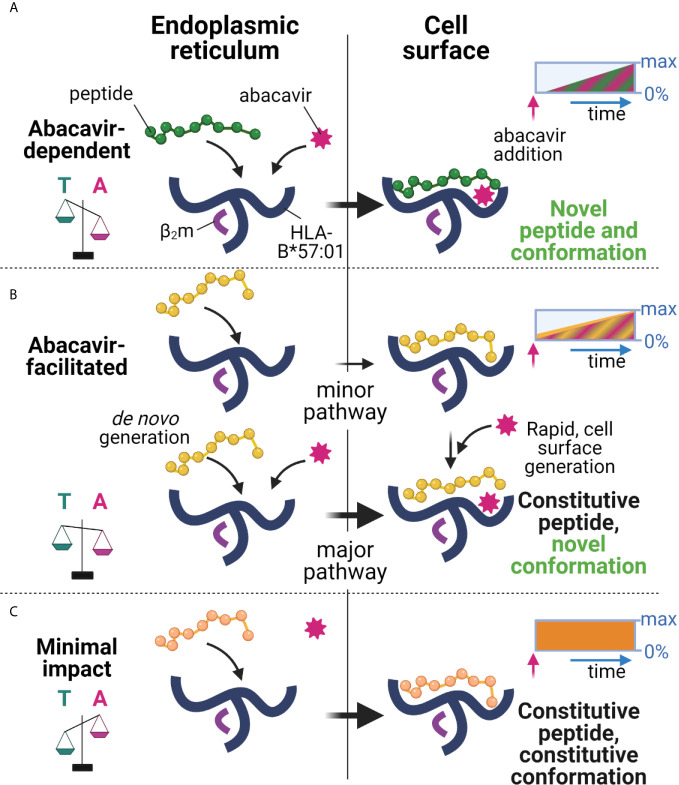
Proposed model of peptide-loading of HLA-B*57:01 in the presence of abacavir. **(A)** When abacavir is present, abacavir-dependent peptides are loaded into HLA-B*57:01 within the endoplasmic reticulum (left). For the abacavir-dependent peptides in our panel, loading was highly dependent on abacavir as compared to tapasin, represented by the scales tipping towards abacavir (magenta, A), as opposed to tapasin (teal, T). After a slight delay consistent with *de novo* complex generation in the ER and progression through the secretory pathway, this generates conformationally novel HLA–abacavir–peptide complexes at the cell surface (right, novel self-peptide and novel conformation). The inset box represents the increasing appearance of cell surface complexes (0%–max) incorporating abacavir-dependent peptides (green) and abacavir (magenta hatching) over time (blue arrow) after abacavir addition (pink arrow), until a maximum is reached (not to scale). **(B)** Abacavir-facilitated peptides are part of the constitutive immunopeptidome (upper panel). For the abacavir-facilitated peptides in our panel, tapasin aids loading in the endoplasmic reticulum in the absence of abacavir, and peptide-loaded HLA traffic to the cell surface. On addition of abacavir, abacavir can load into these HLA-peptide complexes at the cell surface generating novel conformations/stabilising the structure (constitutive peptide, novel conformation), contributing immediately to cellular immunogenicity. In addition, *de novo* generation of HLA-abacavir-peptide complexes occurs within the endoplasmic reticulum, with both abacavir and tapasin promoting peptide binding (lower panel). As such abacavir-facilitated peptides are present in the immunopeptidome prior to abacavir addition (inset box, yellow), with immediate loading of abacavir at the cell surface as well as during *de novo* complex generation, contributing an increasing number of abacavir occupied HLA from the time of abacavir addition (inset box, magenta hatching). **(C)** These changes occur against a background of constitutive peptides which maintain a similar contribution to the immunopeptidome regardless of the presence of abacavir (minimal impact, inset box, orange), whilst a proportion of constitutive peptides are reduced in presentation (inhibited, **Figure 1**, not shown here). This figure was created with BioRender.com.

We and others have shown that abacavir exclusively binds within the HLA-B*57:01 antigen binding groove, altering the preference for the C-terminal amino acid residue of the bound peptide, and changing the immunopeptidome in a process dependent on tapasin and the conventional MHC I antigen presentation pathway (23–26). Whilst this perturbation accounts for many of the T cell responses observed against drug treated HLA-B*57:01^+^ cells, the kinetics of abacavir mediated perturbation of the immunopeptidome, and the specific peptide editing function of tapasin has remained largely unexplored. In this study, we found that HLA-B*57:01 expressing cells rapidly acquired the ability to elicit drug-specific responses from drug-responsive T cell lines following the addition of abacavir, and that the magnitude of these responses increased over several hours (**Figure 3**). Importantly the kinetics at which immunogenicity was acquired generally coincided with the rate that newly synthesised thermostable HLA-B*57:01 molecules progress through the secretory pathway (**Figure 4**) and match the rate at which abacavir and abacavir-induced peptides accumulated within the population of HLA-B*57:01–peptide complexes (**Figure 1**). In addition, abacavir-dependent peptides derive from source proteins occupying similar cellular compartments to those of the constitutive repertoire (**Supplementary Table 1**), consistent with a shared loading pathway. This suggests that immune responses are primarily elicited following the appearance of newly synthesised HLA-B*57:01 complexes at the cell surface, that have selected peptides in the ER and transited the secretory pathway in the presence of abacavir, as opposed to abacavir modifying cell surface HLA-B*57:01–peptide complexes (see model **Figure 8**).

However, the fact that abacavir decreased the dissociation of some “constitutive” relatively stable peptide-loaded B*57:01 complexes (such as KTF and TSL, **Figures 7F, G**) highlights the potential for certain cell surface HLA-B*57:01–peptide complexes to be abacavir receptive (see model **Figure 8**), as hypothesised by Yun et al. for HLA-B*58:01 and oxypurinol (59). This provides a possible mechanism for the formation of immunogenic cell surface abacavir-loaded HLA–peptide complexes, available to stimulate reported “immediate” responding abacavir-responsive T cell clones (37). The relative abundance of these complexes might be expected to rise in the presence of abacavir as a result of a reduction in their turnover at the cell surface (KTF and TSL increased in abundance in the immunopeptidome with abacavir treatment, **Figure 1C**), in addition to increased loading in the ER. We conceive two potential modes by which such “constitutive” HLA–peptide complexes might elicit T cell responses: 1) incorporation of abacavir within the antigen binding cleft results in a structurally novel immunogenic HLA–abacavir–peptide complex, potentially leading to an immediate immune response, or 2) incorporation of abacavir within the antigen binding cleft decreases the dissociation rate of specific peptide complexes, resulting in their increased accumulation within the immunopeptidome, eventually breaching a quantitative threshold required to trigger self-reactive T cells that would otherwise “ignore” the low level of cognate ligand. Notably, the impact of abacavir on peptide presentation and immunogenicity appears analogous to that of micropolymorphism. The location of abacavir within the antigen binding cleft mirrors that of buried polymorphic residues between HLA-B*57:01, HLA-B*57:03, and HLA-B*58:01, which not only change the range and quantitative contribution of peptides within the immunopeptidome, but influence peptide presentation through altered bound-peptide conformation upon binding of identical peptides (55). These parallels between abacavir induced changes in peptide presentation and allo-HLA presentation align with previous investigations defining the overlap in functional responses to abacavir and alloreactivity towards HLA-B*58:01 (60). Overall, the peptide specific modulation of the HLA-B57 immunopeptidome by abacavir reconciles the biophysical studies of abacavir induced altered self with functional studies demonstrating a spectrum of kinetics and dose dependence for activation of abacavir-reactive T cell clones (37).

Through our multifaceted analysis, we observed heterogeneity in the sequence and nature of abacavir modulated peptides such that no simple predictive rule could be applied to estimate the ability of abacavir to induce or modulate levels of presentation of given peptide ligands. For example, abacavir enhanced the binding of two canonical peptide ligands to HLA-B*57:01 molecules (VTKKTYEIW and ITTKAISRW), despite these peptides having C-terminal amino acid residues considered ill-suited for abacavir binding (**Figures 5**, **6**). Our analysis of the assembly of HLA-B*57:01 molecules shows that abacavir does not significantly change the maturation kinetics, dependence on tapasin, or thermal-stability profile of HLA-B*57:01–peptide complexes at a population level (**Figure 4**). This suggests abacavir does not change the intrinsic conformational plasticity of the HLA-B*57:01 protein, which has been hypothesised to determine the ability of MHC I molecules to select a high affinity peptide cargo (34, 61, 62). In this model, peptides are suggested to bind to an unstable, open, intermediate state that is also a substrate for tapasin. Abacavir may also bind preferentially to this open, intermediate state of HLA-B*57:01 molecules, remodelling the peptide-receptive F-pocket whilst the remainder of the peptide binding groove is either empty or partially occupied by interactions with the N-terminus of the peptide ligand (63).

Overall, our *in vitro* analysis has illustrated that abacavir-induced peptides likely accrue within the immunopeptidome as a result of their improved binding kinetics in the presence of abacavir, which can be further enhanced by tapasin. In conjunction with our quantitative analysis of the contribution of specific peptides to the immunopeptidome, these data suggest that abacavir diversifies the HLA-B*57:01 immunopeptidome by enabling the stable binding of peptides with aliphatic C-terminal anchors that are either low abundance or absent in the constitutive immunopeptidome. These abacavir-induced peptides potentially bind at the expense of rapidly dissociating peptides, such as RVD, whilst peptides that bind with longer half-lives are less effected. Furthermore, a low level of tapasin independent loading of HLA-B*57:01 molecules was observed, which may allow some abacavir-induced (and constitutive) peptides to enter the immunopeptidome in cellular compartments beyond the ER. Such effects could further explain some of the heterogeneity reported for the kinetics of activation of drug specific T cells (37, 64). Although beyond the scope of the current study, dissection of the contribution of peptides exhibiting different loading characteristics to the immune response, using *in vitro* expanded T cells or through the use of HLA-B*57:01 transgenic mouse models of abacavir hypersensitivity (65), is critical to pinpoint their contribution to drug hypersensitivity.

Overall, our data highlight that the majority of the immunopeptidome perturbation occurs during *de novo* loading of complexes. Collectively, these findings highlight the importance of the ER environment in generation of the immunopeptidome, demonstrating the potential for not only chaperones, but small molecule ligands, to differentially impact the binding and dissociation of distinct peptides to and from assembling MHC I molecules, raising the possibility that alterations in the cellular metabolome may also alter peptide presentation.

## Data Availability Statement

The datasets presented in this study can be found in online repositories. The names of the repository/repositories and accession number(s) can be found below: ProteomeXchange Consortium via the PRIDE (44) partner repository, https://www.ebi.ac.uk/pride/archive/, PXD024331.

## Ethics Statement

The studies involving human participants were reviewed and approved by Monash University (HREC 4717 & 2297) and the Australian Bone Marrow Donor Registry (2013/04). The patients/participants provided their written informed consent to participate in this study.

## Author Contributions

PI, AvH, TE, and AP designed the experiments. PI, AvH, RD, NC, NM, and SK performed the experiments. LK, MB, and JM provided the reagents. PI, AvH, RD, NC, NM, SK, TE, and AP analyzed the data. PI, AvH, TE, and AP wrote the manuscript. All authors contributed to the article and approved the submitted version.

## Funding

This work was funded by the National Health and Medical Research Council Australia (NHMRC) Project grants 1122099 and 1165490 (awarded to AP) and by a Cancer Research UK programme grant C7056A and BBSRC project grant BB/L010402/1 (both awarded to TE). PI was supported by a NHMRC Early Career Fellowship (1072159) and a Monash University Faculty of Medicine, Nursing and Health Sciences Senior Postdoctoral Fellowship. AP acknowledges salary support from a NHMRC Principal Research Fellowship (1137739).

## Conflict of Interest

The authors declare that the research was conducted in the absence of any commercial or financial relationships that could be construed as a potential conflict of interest.

## Abbreviations

MHC I, Major histocompatibility complex class I; CTLs, cytotoxic T lymphocytes; HLA, Human Leukocyte Antigen; ER, Endoplasmic reticulum; PBMCs, peripheral blood mononuclear cells; LC-MS/MS, liquid chromatography-tandem mass spectrometry; LC-MRM-MS, liquid chromatography-multiple reaction monitoring-mass spectrometry; *β*_2_m, *β*_2_-microglobulin.
