# Supplementary material for: Kinetics of Abacavir-Induced Remodelling of the Major Histocompatibility Complex Class I Peptide Repertoire
Source: Front Immunol. 2021 May 19;12:672737. doi: 10.3389/fimmu.2021.672737 (PMC8170132; doi:10.3389/fimmu.2021.672737)
Supplement: Supplementary file 1 [file DataSheet_1.docx]

Supplementary Material

# Supplementary Methods

## MHC I cell surface expression and stability assays

To measure HLA-B*57:01 surface expression levels: 60 µM abacavir or PBS was added to 24 well plates containing 721.220.B*57:01 or 721.220.B*57:01.tapasin cells at 3x10^5^ cells/mL and cultured for 20 hrs at 37 °C in 5% CO_2_. The cells were then washed with PBS, split into two and stained with W6/32 followed by goat anti-mouse immunoglobulin conjugated with FITC (Sigma) or FITC alone and analysed by FACS (BD FACSCaliburTM, BD Biosciences). To determine if abacavir was able to stabilise HLA-B*57:01 molecules expressed on the surface of various cell types: 60 µM abacavir or PBS was added to 24 well plates containing cells at 3x10^5^ cells/mL and cultured for 20 hrs at 37 °C in 5% CO_2_. Brefeldin A (Sigma) was added at 5 µg/mL for the specified time points. At the end of the time course each well was washed with PBS, split into two and stained as above. Surface MHC I was expressed as the percentage of mean channel fluorescence at time point 0.

# Supplementary Figures
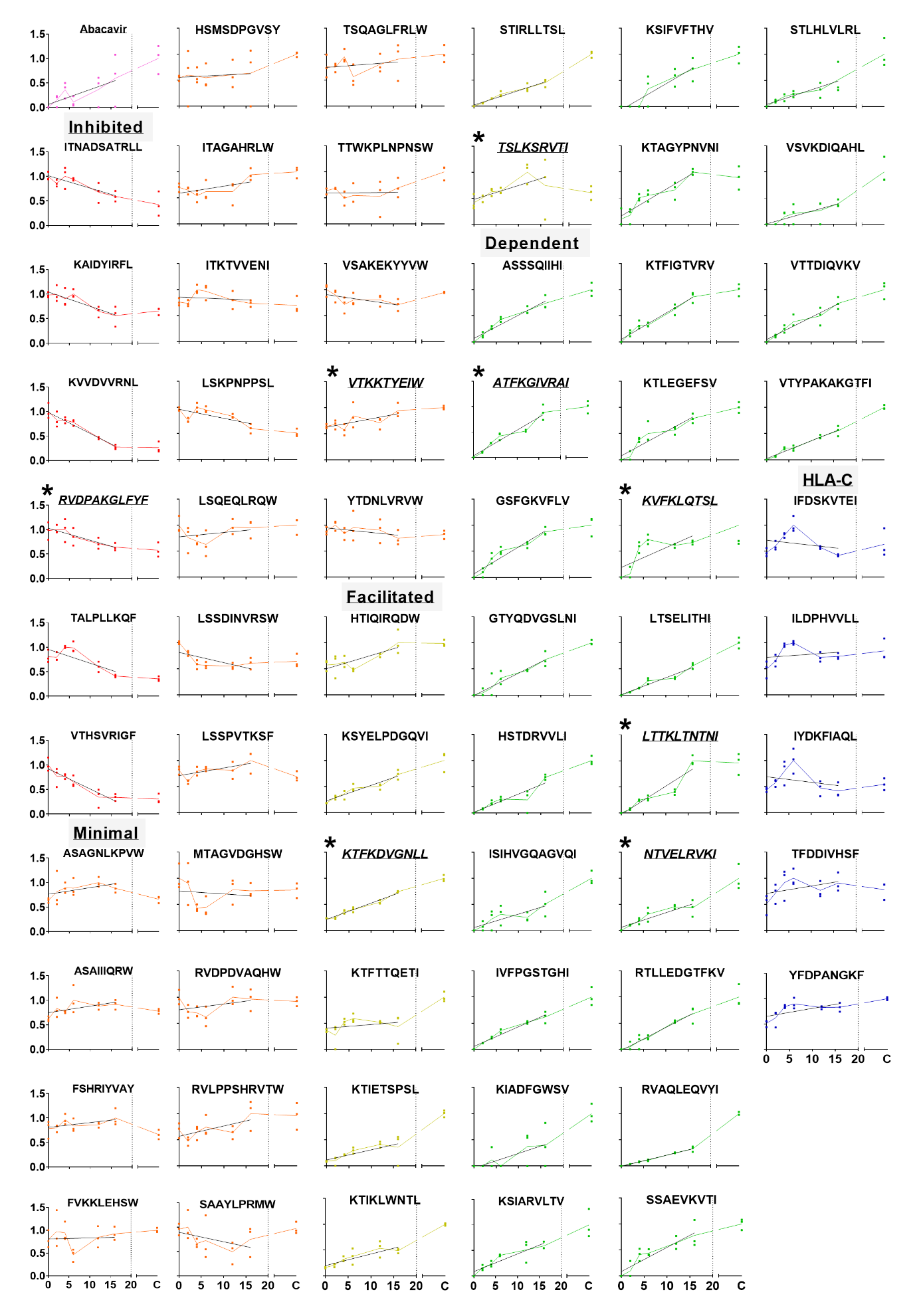


**Supplementary figure 1. Abacavir perturbs the HLA-B*57:01 immunopeptidome by increasing or decreasing the contribution of distinct subsets of peptides.** Data from Figure 1 with each peptide/abacavir plotted on individual axes. Squares denote individual data points, with a coloured line showing the mean, coloured according to the category assigned based on the linear regression across the first 16 hrs abacavir exposure as described in the methods. The black line shows the linear regression. The duration of exposure to abacavir is shown in hours on the x axis, where “C” denotes constant abacavir exposure. Peptide abundance is scaled to maximum mean for each peptide and is shown on the y axis. Asterisks denote peptides assayed in *in vitro* peptide binding and dissociation experiments. Colours are as follows: Abacavir (pink), Inhibited (red), Minimal impact (orange), Facilitated (yellow), Dependent (green), HLA-C (blue).


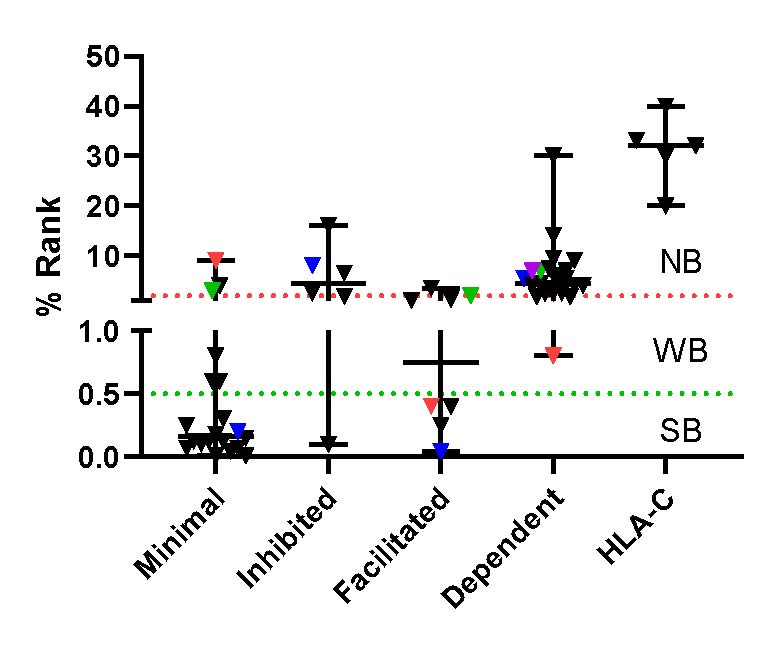


**Supplementary figure 2. Abacavir dependent peptides are predicted non-binders of HLA-B*57:01.** Binding predictions for HLA-B*57:01 were performed using netMHC4.0 (1, 2), and are reported as a % Rank. Default thresholds for strong binders (SB, % Rank < 0.5, green dotted line), weak binders (WB, % Rank < 2, red dotted line) and non-binders (NB, % Rank >2) were employed. Each point represents the predicted binding of a single peptide within the category (as in Supplementary table 1). Median and range are depicted for each category. Selected peptides are indicated with coloured symbols as follows - Minimal: VTKKTYEIW (blue); ITKTVVENI (red); LSKPNPPSL (green); Inhibited: RVDPAKGLFYF (blue); Facilitated: HTIQIRQDW (blue); KTFKDVGNLL (red); TSLKSRVTI (green); Dependent: ATFKGIVRAI (blue); KVFKLQTSL (red); LTTKLTNTNI (green); NTVELRVKI (purple).


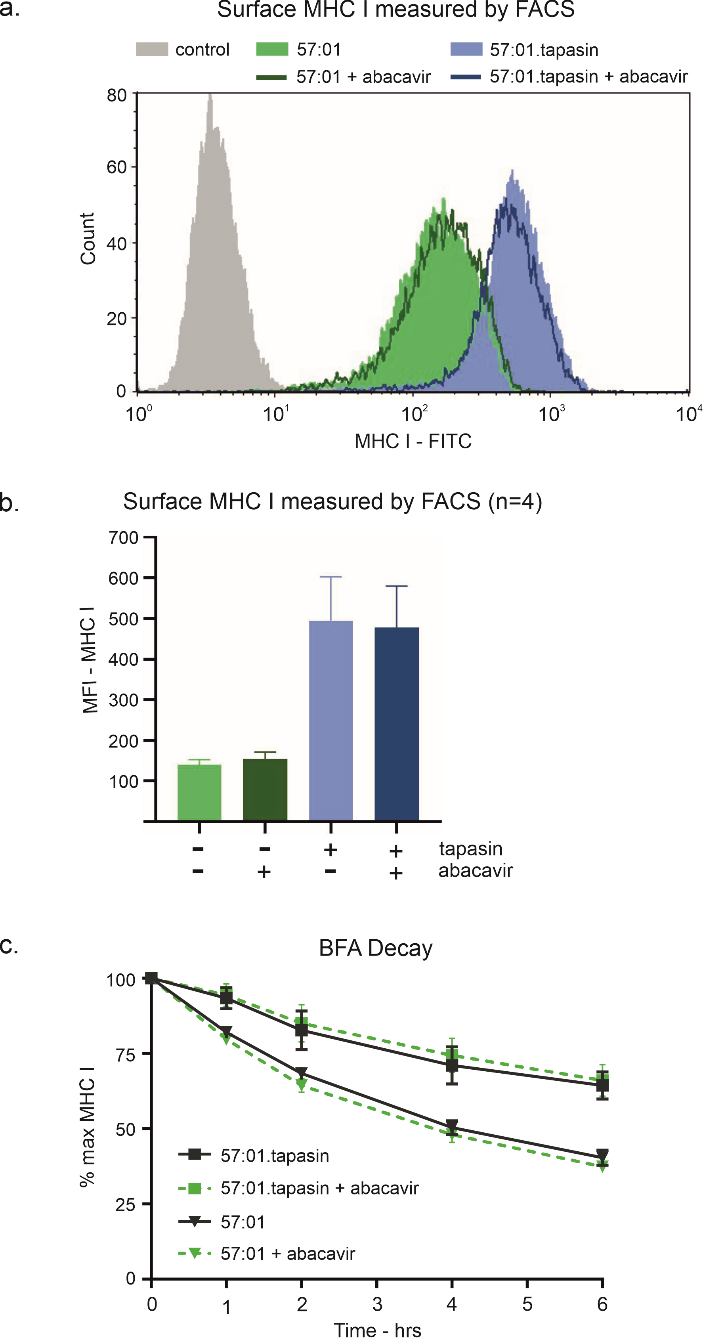


**Supplementary figure 3. Tapasin, but not abacavir, improves the expression level and stability of HLA-B*57:01 on the cell surface.** 721.220.B*57:01 or 721.220.B*57:01.tapasin cells were cultured with 60 µM abacavir or PBS for 20 hrs before the cells were stained with W6/32 antibody and goat anti-mouse immunoglobulin-FITC labelled antibody and analysed by flow cytometry. **(A)** Histogram showing HLA-B*57:01 expression from a representative experiment. Control represents cells stained with goat anti-mouse immunoglobulin-FITC labelled antibody alone. **(B)** Bar chart showing the mean average HLA-B*57:01 expression from four independent experiments, with the standard deviation shown as coloured vertical lines edged with horizontal bars. **(C)** Line graph showing stability of surface expressed HLA-B*57:01 molecules. Cells were cultured with 60 µM abacavir or PBS for 20 hrs before Brefeldin A was added at 5 µg/mL for the last 0-6 hrs of culture as specified. At the end of the time course cells were stained with W6/32 antibody and goat anti-mouse immunoglobulin-FITC labelled antibody and analysed by flow cytometry. HLA-B*57:01 surface expression is shown as the percentage of mean fluorescence at time point 0. Values shown are the mean of three individual experiments, error bars indicate the standard deviation.

# Supplementary Table

| **Peptide** | **Condition** | **Half life (hours)** | **Standard deviation** | **Replicates** |
| --- | --- | --- | --- | --- |
| VTKK*TYEIW | Comp | 374.80 | 40.65 | 3 |
|  | Comp+Abacavir | 373.30 | 23.28 |  |
|  | Comp+Tapasin | 207.90 | 86.67 |  |
|  | Comp+Tapasin+Abacavir | 198.40 | 51.05 |  |
| RVDPAK*GLFYF | Comp | 0.31 | 0.06 | 4 |
|  | Comp+Abacavir | 0.26 | 0.02 |  |
|  | Comp+Tapasin | 0.35 | 0.04 |  |
|  | Comp+Tapasin+Abacavir | 0.26 | 0.03 |  |
| KVFK*LQTSL | Comp | 3.27 | 2.55 | 4 |
|  | Comp+Abacavir | 51.83 | 9.49 |  |
|  | Comp+Tapasin | 1.06 | 2.48 |  |
|  | Comp+Tapasin+Abacavir | 2.47 | 6.36 |  |
| NTVELRVK*I | Comp | 2.67 | 0.87 | 3 |
|  | Comp+Abacavir | 233.60 | 80.55 |  |
|  | Comp+Tapasin | 1.60 | 0.76 |  |
|  | Comp+Tapasin+Abacavir | 142.70 | 23.81 |  |
| ATFK*GIVRAI | Comp | 2.34 | 0.29 | 3 |
|  | Comp+Abacavir | 71.63 | 9.87 |  |
|  | Comp+Tapasin | 2.04 | 0.38 |  |
|  | Comp+Tapasin+Abacavir | 33.59 | 13.36 |  |
| KTFK*DVGNLL | Comp | 402.30 | 199.25 | 3 |
|  | Comp+Abacavir | 814.20 | 479.93 |  |
|  | Comp+Tapasin | 4.97 | 52.62 |  |
|  | Comp+Tapasin+Abacavir | 62.89 | 17.00 |  |
| TSLK*SRVTI | Comp | 7.41 | 1.83 | 3 |
|  | Comp+Abacavir | 22.41 | 9.52 |  |
|  | Comp+Tapasin | 5.12 | 2.50 |  |
|  | Comp+Tapasin+Abacavir | 10.38 | 4.59 |  |
| ITTK*AISRW | Comp | 35.63 | 6.16 | 3 |
|  | Comp+Abacavir | 35.07 | 4.61 |  |
|  | Comp+Tapasin | 14.20 | 3.66 |  |
|  | Comp+Tapasin+Abacavir | 12.58 | 2.98 |  |

**Supplementary Table 2. Dissociation rates of TAMRA-labelled peptides from HLA-B*57:01fos molecules.** The rates at which the indicated peptides dissociated from HLA-B*57:01fos molecules in the presence of excess unlabelled competing peptide (“Comp”), abacavir and tapasin (data shown in figure 7) was measured by Graphpad Prism v9.1.0. The dissociation rates were calculated by performing non-linear regression, using the one-phase decay method, with plateaus constrained to be equal to 50 mP (apart from being unconstrained for RVD peptide). The number of experimental replicates, and the standard deviation in the measured dissociation rates across the replicates is also presented.

# Supplementary References

1. Andreatta M, Nielsen M. Gapped sequence alignment using artificial neural networks: application to the MHC class I system. Bioinformatics. 2016;32(4):511-7.

2. Nielsen M, Lundegaard C, Worning P, Lauemoller SL, Lamberth K, Buus S, et al. Reliable prediction of T-cell epitopes using neural networks with novel sequence representations. Protein science : a publication of the Protein Society. 2003;12(5):1007-17.
